# Genome-wide analysis of *MdPLATZ* genes and their expression during axillary bud outgrowth in apple (*Malus domestica* Borkh.)

**DOI:** 10.1186/s12864-023-09399-x

**Published:** 2023-06-15

**Authors:** Jiuyang Li, Yongliang Zhao, Yaohui Zhang, Feng Ye, Zhengcun Hou, Yuhang Zhang, Longjie Hao, Guofang Li, Jianzhu Shao, Ming Tan

**Affiliations:** College of Horticulture, Hebei Agricultural University, Hebei, 071000 China

**Keywords:** Apple, *PLATZ* transcription factor, Gene expression, Axillary bud outgrowth, Shoot branching

## Abstract

**Background:**

Branching is a plastic character that affects plant architecture and spatial structure. The trait is controlled by a variety of plant hormones through coordination with environmental signals. Plant AT-rich sequence and zinc-binding protein (PLATZ) is a transcription factor that plays an important role in plant growth and development. However, systematic research on the role of the PLATZ family in apple branching has not been conducted previously.

**Results:**

In this study, a total of 17 *PLATZ* genes were identified and characterized from the apple genome. The 83 PLATZ proteins from apple, tomato, *Arabidopsis*, rice, and maize were classified into three groups based on the topological structure of the phylogenetic tree. The phylogenetic relationships, conserved motifs, gene structure, regulatory *cis*-acting elements, and *microRNAs* of the *MdPLATZ* family members were predicted. Expression analysis revealed that *MdPLATZ* genes exhibited distinct expression patterns in different tissues. The expression patterns of the *MdPLATZ* genes were systematically investigated in response to treatments that impact apple branching [thidazuron (TDZ) and decapitation]. The expression of *MdPLATZ*1, 6, 7, 8, 9, 15, and 16 was regulated during axillary bud outgrowth based on RNA-sequencing data obtained from apple axillary buds treated by decapitation or exogenous TDZ application. Quantitative real-time PCR analysis showed that *MdPLATZ6* was strongly downregulated in response to the TDZ and decapitation treatments, however, *MdPLATZ15* was significantly upregulated in response to TDZ, but exhibited little response to decapitation. Furthermore, the co-expression network showed that PLATZ might be involved in shoot branching by regulating branching-related genes or mediating cytokinin or auxin pathway.

**Conclusion:**

The results provide valuable information for further functional investigation of *MdPLATZ* genes in the control of axillary bud outgrowth in apple.

**Supplementary Information:**

The online version contains supplementary material available at 10.1186/s12864-023-09399-x.

## Background

Transcription factors (TFs) are proteins that specifically bind to *cis*-acting elements in promoter regions and thereby regulate gene expression [1]. They play important roles in diverse life processes, such as regulation of plant growth and development, defense against stress, and signal transduction [2]. In recent years, increased research attention has been focused on TFs.

Plant AT-rich sequence and zinc-binding protein (PLATZ) is a TF, first identified in pea, that is composed of two zinc finger domains in pea: C-X2-H-X11-C-X2-C-X (4–5)-C-X2-C-X (3–7)-H-X2-H and C-X2-C-X (10–11)-C-X3-C (X represents any amino acid) [3]. In recent years, *PLATZ* TFs have been identified in many plant species; for example, 12 *PLATZ* family members have been identified in *Arabidopsis* [4], 17 members in maize [5], 15 members in rice [4], 62 members in wheat [6], and 86 members in *Malus* [7].

Many studies have confirmed that PLATZ TFs are associated with the regulation of plant growth and development as well as response to abiotic stresses [3, 8]. In maize, the *PLATZ* gene *FL3* is involved in regulating the transcription of tRNA and 5S rRNA during endosperm development and flowering [9]. In rice, *GL6* has been identified as a *PLATZ* gene and functions in regulating the length and number of rice panicles [10]. ORESARA 15 an *Arabidopsis* PLATZ TF, promotes the rate and duration of leaf cell proliferation and inhibits leaf senescence. In *Arabidopsis*, *PLATZ1* and *PLATZ2* play important roles in tolerance of seed dehydration [11], and *PLATZ2* can also affect plant sensitivity to salt stress [12]. Studies on sugarcane show that a *PLATZ* gene may be a transcriptional regulator of secondary cell wall synthesis [13].

Shoot branching is a quality trait important in the establishment of plant architecture. The planting of ideally branched nursery trees is crucial in modern apple orchards [14, 15]. The lateral buds on branches generally develop into flower buds in the first year, and contribute to fruit number and quality in the succeeding year [16]. The highest yield in initial and subsequent stages of orchard management can be achieved with nursery trees with an ideal branch architecture. However, different treatments are applied in nurseries to increase the number of branches, such as application of exogenous hormones [e.g., 6-benzylaminopurine (6-BA), or thidazuron (TDZ)] or pruning methods. Therefore, understanding the molecular mechanism responsible for apple shoot branching would be greatly beneficial.

The axillary buds of apple, located in the leaf axils, are generally dormant owing to the correlative inhibition exerted by apical buds [17]. Outgrowth of axillary buds (i.e., branching) is regulated by multiple endogenous and exogenous factors [18–20]. Previous studies have demonstrated that auxin indirectly inhibits branching [21, 22]. Cytokinin (CK) stimulates the outgrowth of axillary buds directly [23, 24]. Our recent study showed that exogenous CK and decapitation can induce the activation and outgrowth of axillary buds in apple, and suppression of CK synthesis restricts the outgrowth of axillary buds induced by decapitation [25]. Spring budburst in apple is specifically triggered by CKs from the shoot [26]. Strigolactone (SL), a secondary messenger of auxin that directly inhibits axillary bud outgrowth [27], is reported to act antagonistically with CK in regulating bud outgrowth [28]. In addition to hormone signals, the control of functional genes contributes to the regulation of bud activation. There is evidence that *PLATZ* is involved in the transition from primary growth to secondary growth during bud development in poplar [29], which provides a clue to the role of *PLATZ* genes in the control of bud growth. However, there is little corresponding research on apple.

In the present study, we identified the *MdPLATZ* family members in the entire apple genome, characterized their basic properties, and explored their phylogenetic relationships and expression patterns. To clarify the role of *MdPLATZ* genes in shoot branching in apple, we investigated their expression patterns in treated axillary buds using transcriptomic data. Selected *MdPLATZ* genes were further analyzed in response to TDZ and decapitation treatments by quantitative real-time PCR (qRT-PCR) analysis, and were also used for interaction network analysis. Changes in gene expression in response to the branching-related treatments and prediction of proteins that interact with MdPLATZ proteins provided evidence for their potential roles in apple bud outgrowth. The results provide a foundation for further analysis of the functional role of *MdPLATZ* genes in apple shoot branching.

## Results

### Identification of *MdPLATZ* gene family members in apple

**Fig. 1:**
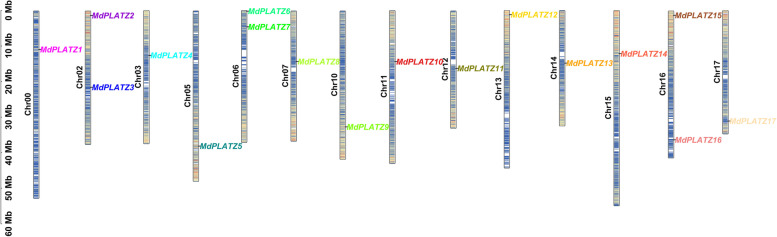
Chromosome location of apple *MdPLATZ* genes. The colors on the chromosomes represent the gene density. Blue: smaller gene density, red: greater gene density. Mb: chromosome length, 1 Mb = 1,000,000 bp. The apple chromosomes shown in Apple GDDH13_v1.1 genome are of the 17 types chr01-17, however, there are 801 contigs assembled into one pseudomolecule that could not be assigned to a chromosome, which named Chr00

A total of 17 *MdPLATZ* genes were identified from the apple genome database (GDDH13.1–1). The *MdPLATZ* genes were named based on their chromosomal location (Table 1). The sequence lengths of the MdPLATZ proteins ranged from 147 (MdPLATZ17) to 257 (MdPLATZ2) amino acids and, accordingly, the molecular weight varied from 17.23 to 29.40 kDa. The theoretical isoelectric point (pI) of all 17 MdPLATZ proteins was more than 7. All proteins had a grand average hydropathicity values of less than zero, suggesting that they were all hydrophilic proteins. The instability index of all MdPLATZ members was more than 40, indicating that they are unstable proteins. The MdPLATZ proteins were predicted to be localized in the nucleus, except for three proteins: MdPLATZ5 (localized to chloroplasts), and MdPLATZ6 and MdPLATZ9 (localized to chloroplasts and the nucleus). The reliability of the tertiary structure of all MdPLATZ proteins was greater than 80% (Supplementary Fig. S1). Detailed information on the protein secondary structure is listed in Table S2. The 17 *MdPLATZ* genes were distributed on 14 chromosomes (Chr) (Fig. 1). Chromosomes 02, Chr06, and Chr16 contained the highest number with two *MdPLATZ* genes whereas Chr00, Chr03, Chr05, Chr07, Chr10, Chr11, Chr12, Chr13, Chr14, Chr15, and Chr17 each carried only one *MdPLATZ* gene.

**Table 1:** Information on *MdPLATZ* gene family members in apple

Gene name	Gene ID	Amino acids (aa)	pI	Molecular mass (kD)	Instability index	Grand average of hydropathicity	Subcellular localization
*MdPLATZ1*	*MD00G1059100*	252	8.55	28.54	55.19	-0.496	Nucleus
*MdPLATZ2*	*MD02G1017000*	257	8.56	29.39	59.17	-0.496	Nucleus
*MdPLATZ3*	*MD02G1208800*	221	8.82	24.67	58.34	-0.239	Nucleus
*MdPLATZ4*	*MD03G1129200*	192	9.51	21.97	64.26	-0.843	Nucleus
*MdPLATZ5*	*MD05G1248500*	254	8.39	28.49	57.66	-0.355	Chloroplast
*MdPLATZ6*	*MD06G1001300*	252	8.78	28.44	51.01	-0.515	Chloroplast. Nucleus
*MdPLATZ7*	*MD06G1035500*	222	9.25	24.88	49.62	-0.698	Nucleus
*MdPLATZ8*	*MD07G1117000*	245	8.81	27.39	52.81	-0.189	Nucleus
*MdPLATZ9*	*MD10G1229000*	255	8.39	28.55	56.28	-0.408	Chloroplast. Nucleus
*MdPLATZ10*	*MD11G1151300*	212	9.3	24.31	68.23	-0.695	Nucleus
*MdPLATZ11*	*MD12G1103000*	197	8.88	22.04	59.31	-0.514	Nucleus
*MdPLATZ12*	*MD13G1017800*	214	9.5	24.71	46.55	-0.249	Nucleus
*MdPLATZ13*	*MD14G1097400*	197	8.52	22.00	64.2	-0.458	Nucleus
*MdPLATZ14*	*MD15G1161500*	255	8.75	29.10	62.78	-0.442	Nucleus
*MdPLATZ15*	*MD16G1015800*	227	9.02	25.76	46.5	-0.423	Nucleus
*MdPLATZ16*	*MD16G1273400*	226	9.33	25.26	48.58	-0.687	Nucleus
*MdPLATZ17*	*MD17G1254800*	147	8.24	17.23	43.3	-0.916	Nucleus

### Phylogenetic relationships and gene structure analysis of MdPLATZ family members

**Fig. 2:**
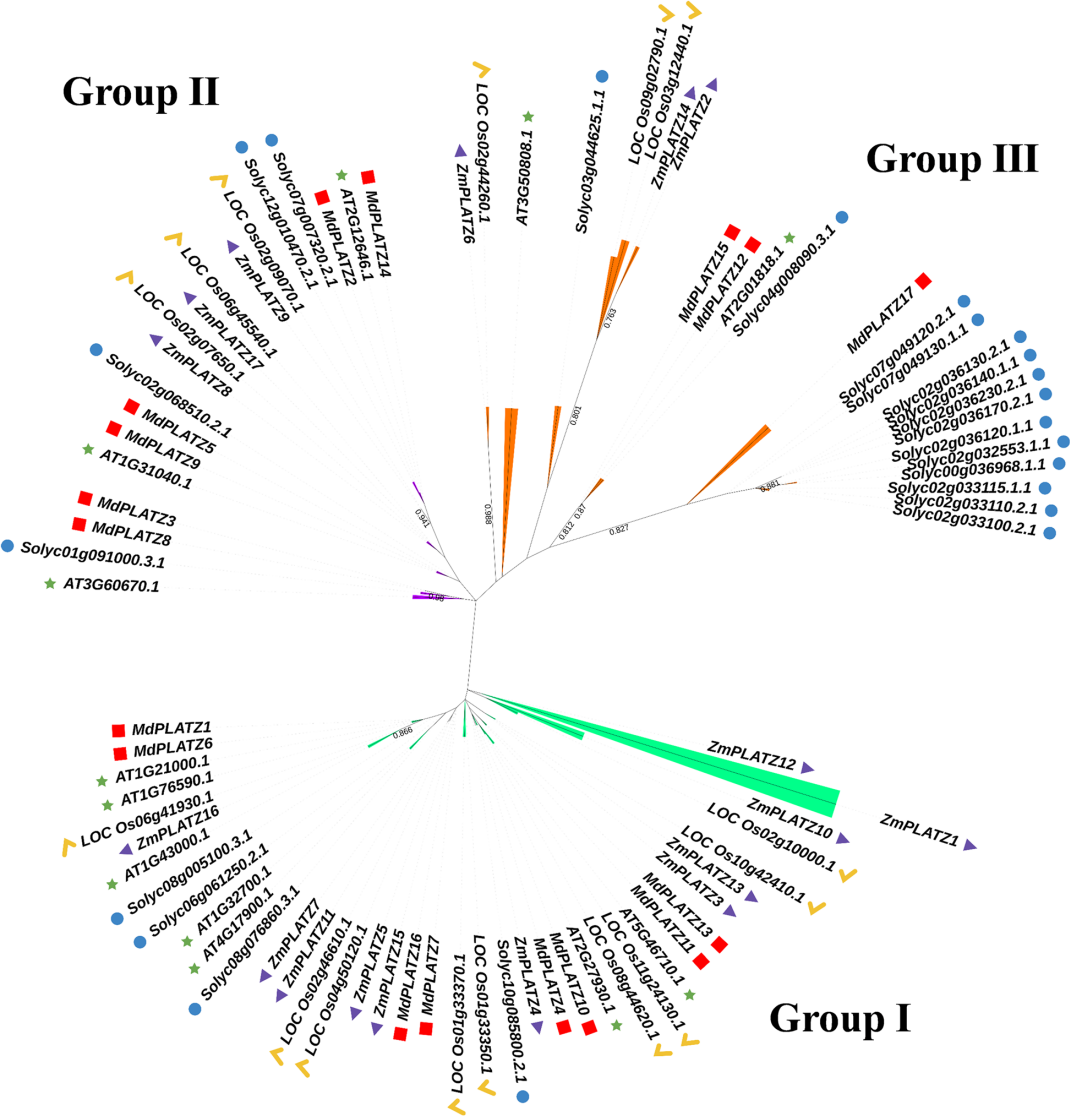
Phylogenetic tree of PLATZ genes from apple, tomato and *Arabidopsis*, rice, and maize. A red box, blue circle, green star, purple check, and brown triangle represent the PLATZ proteins from apple, tomato, *Arabidopsis*, rice, and maize, respectively. Groups I to III are indicated by different colors

To explore the evolution of the MdPLATZ proteins, a phylogenetic tree of 83 PLATZ members, comprising those from apple (17 members), tomato (22 members), *Arabidopsis* (12 members), rice (15 members), and maize (17 members), was constructed (Fig. 2). The PLATZs proteins were classified into three subfamilies (Groups I to III) based on the topology of the phylogenetic tree. The MdPLATZ proteins were distributed in all three groups. Among the three groups, the apple PLATZ proteins were mainly aggregated in Groups I and II, suggesting that these PLATZ genes were conserved. MdPLATZ17 was only clustered with tomato PLATZ proteins in Group III (Fig. 2). In addition, most apple PLATZs shared sister clades with *Arabidopsis* or tomato PLATZs.

**Fig. 3:**
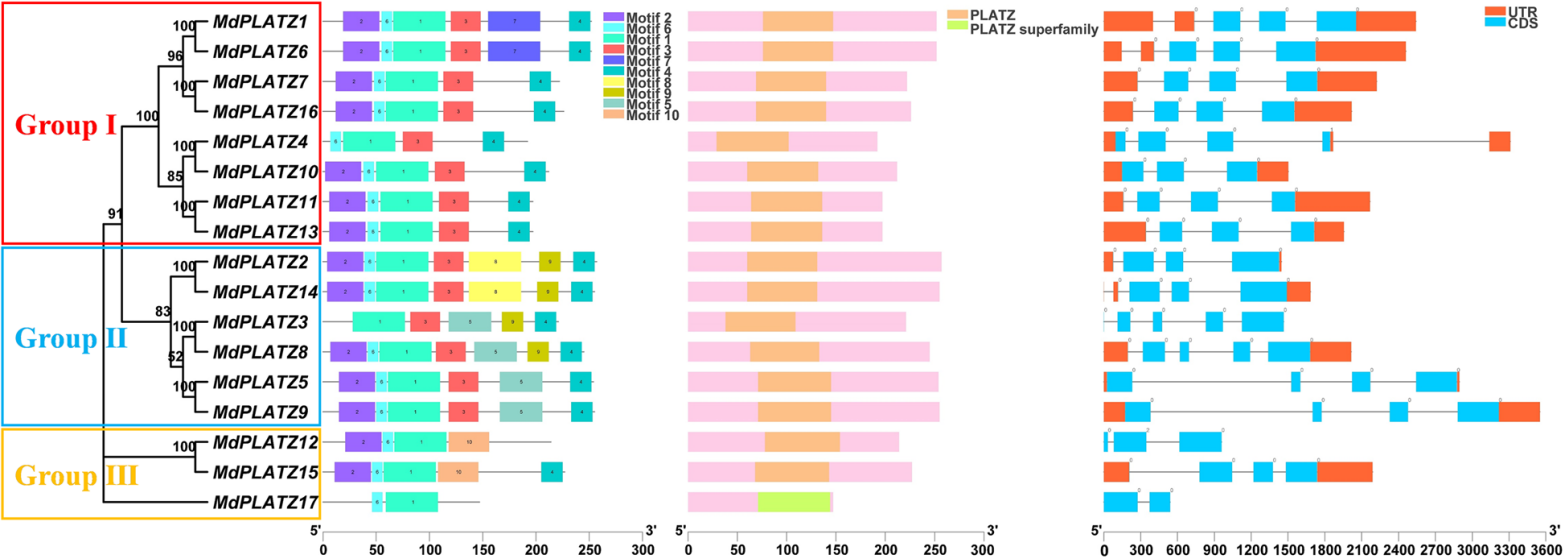
Gene structure and conserved motif analysies of MdPLATZ family members. **A** Phylogenetic tree of MdPLATZ proteins. The numbers on the evolutionary tree represent the bootstrap values. The larger the value, the higher the credibility, while the smaller the value, the lower the credibility. **B** Distribution of conserved motifs in MdPLATZ proteins. Boxes of different colors represent the ten putative motifs. **C** Distribution of conserved domains in MdPLATZ proteins. **D** Exon/intron structure of *MdPLATZ* genes

The 17 apple MdPLATZs were clustered in Groups I to III (Fig. 3A). Protein structure analysis of the *MdPLATZ* family members identified ten regular motifs (Fig. 3B). All MdPLATZ proteins contained motif1, and most members also contained motif2, 4, and 6. Except for the MdPLATZ proteins of Group III, all proteins contained motif3. A total of six MdPLATZ proteins contained motif5, 8, and 9, which were only detected in the proteins of Group II. Only MdPLATZ12 and MdPLATZ15 (Group III) contained motif10. All MdPLATZ proteins contained the PLATZ conserved domain (Fig. 3C). Two zinc finger domains, C-X2-H-X11-C-X2-C-X(4–5)-C-X2-C-X(3–7)-H-X2-H and C-X2-C-X(10–11)-C-X3-C), were present in the MdPLATZ primary amino acid sequence except in MdPLATZ3 and MdPLATZ4, which contained only the C-X2-C-X(10–11)-C-X3-C domain (Supplementary Fig. S2). The gene structures show that the *MdPLATZs* in Group I contain 4 CDS, except for *MdPLATZ10* which has 3 CDS. The *MdPLATZs* of Group II mostly contain 4 CDS, and only *MdPLATZ3* and *MdPLATZ8* contain 5 CDS. The *MdPLATZs* of Group III contain more than 1 CDS. Except for *MdPLATZ17*, which contains 2 exons, all other *MdPLATZ* family members contain more than 2 exons (Fig. 3D). Only *MdPLATZ3*, *MdPLATZ12*, and *MdPLATZ17* did not contain an UTR (Fig. 3D).

### Synteny analysis of the *MdPLATZ* gene family

**Fig. 4:**
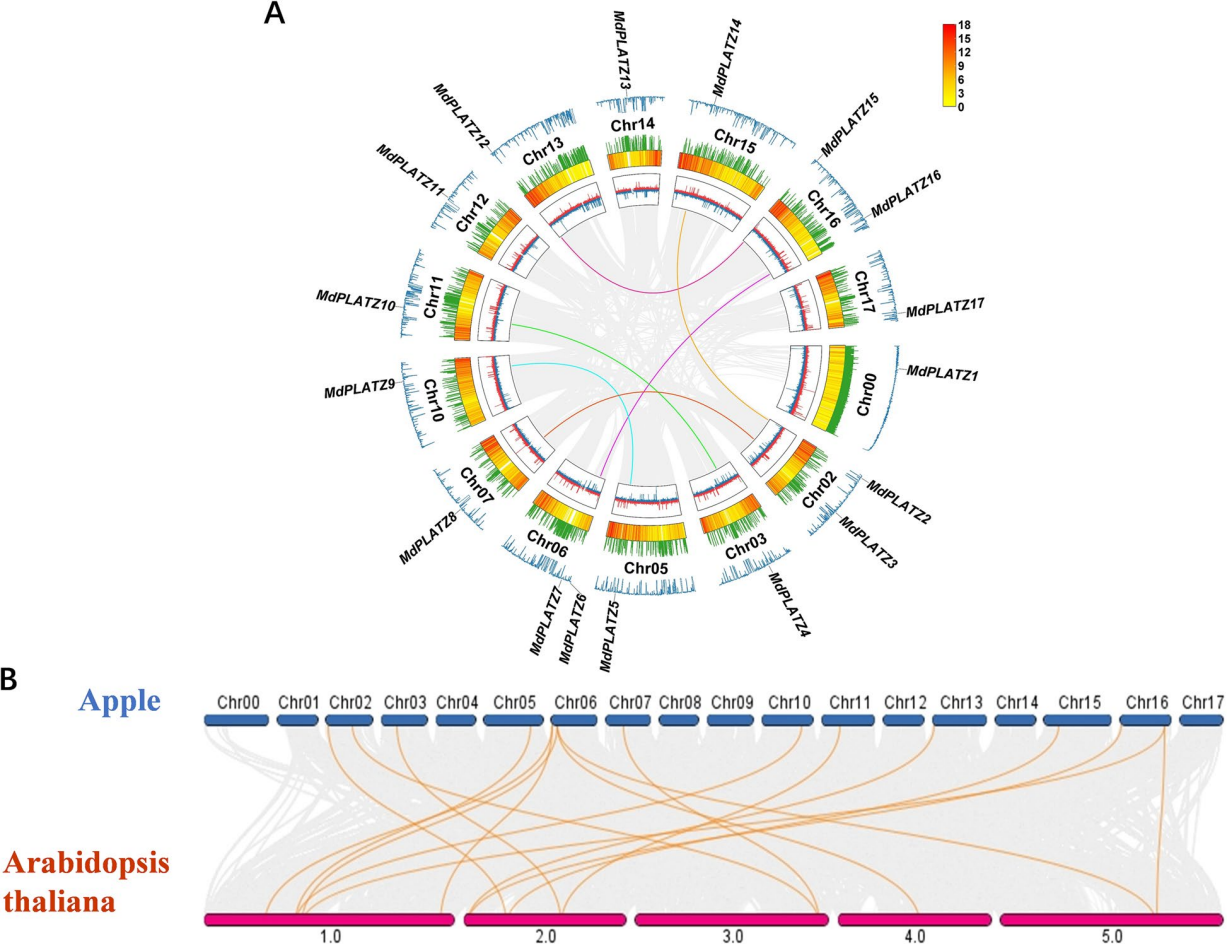
Synteny analysis of *PLATZ* genes in apple and *Arabidopsis*. **A** Gene location, duplication, and synteny analysis of *MdPLATZ* genes. The gray curves indicate the collinear regions in the apple genome and the colored curves indicate the gene pairs that have undergone segmental duplication. From the inside out, the first circle is GC skew, the second circle is gene density, the third circle is N-ratio, and the fourth circle is GC ratio. The color scale represents the number of genes per 100,000 bin on a chromosome. Red represents a high number of genes, while yellow represents a low number of genes. **B** Synteny analysis of *PLATZ* genes between apple and *Arabidopsis*. The gray lines in the background indicate the syntenic blocks between species. The collinear gene pairs are linked with orange lines

The 17 identified *MdPLATZ* genes were distributed on 14 of the 17 apple chromosomes. To analyze the evolution of the *MdPLATZ* gene family in apple, gene segmental and tandem duplication events were analyzed (Fig. 4A, Supplementary Table S3). Segmental duplications were identified as homologs on different chromosomes. Six pairs of segmental duplicates were detected among the 17 *MdPLATZ* genes: *MdPLATZ2/MdPLATZ14*, *MdPLATZ3/MdPLATZ8*, *MdPLATZ4/MdPLATZ10*, *MdPLATZ5/MdPLATZ9*, *MdPLATZ7/MdPLATZ16*, and *MdPLATZ12/MdPLATZ15*. No *MdPLATZ* genes were derived from tandem duplication events. The *Ka/Ks* ratio of the duplicated gene pairs ranged from 0.08 to 0.60 (less than 1) (Supplementary Table S4), suggesting that purifying selective pressure occurred during *MdPLATZ* gene family evolution and a conserved function may be shared among these genes. In addition, a comparative syntenic map between the apple and *Arabidopsis* genomes was constructed. As shown in Fig. 4B, 13 apple *MdPLATZ* genes were collinear with 10 *AtPLATZ* genes in *Arabidopsis*, suggesting that these orthologous pairs may be important for plant evolution.

### Identification of *cis*-acting elements in the promoter of *MdPLATZ* genes

**Fig. 5:**
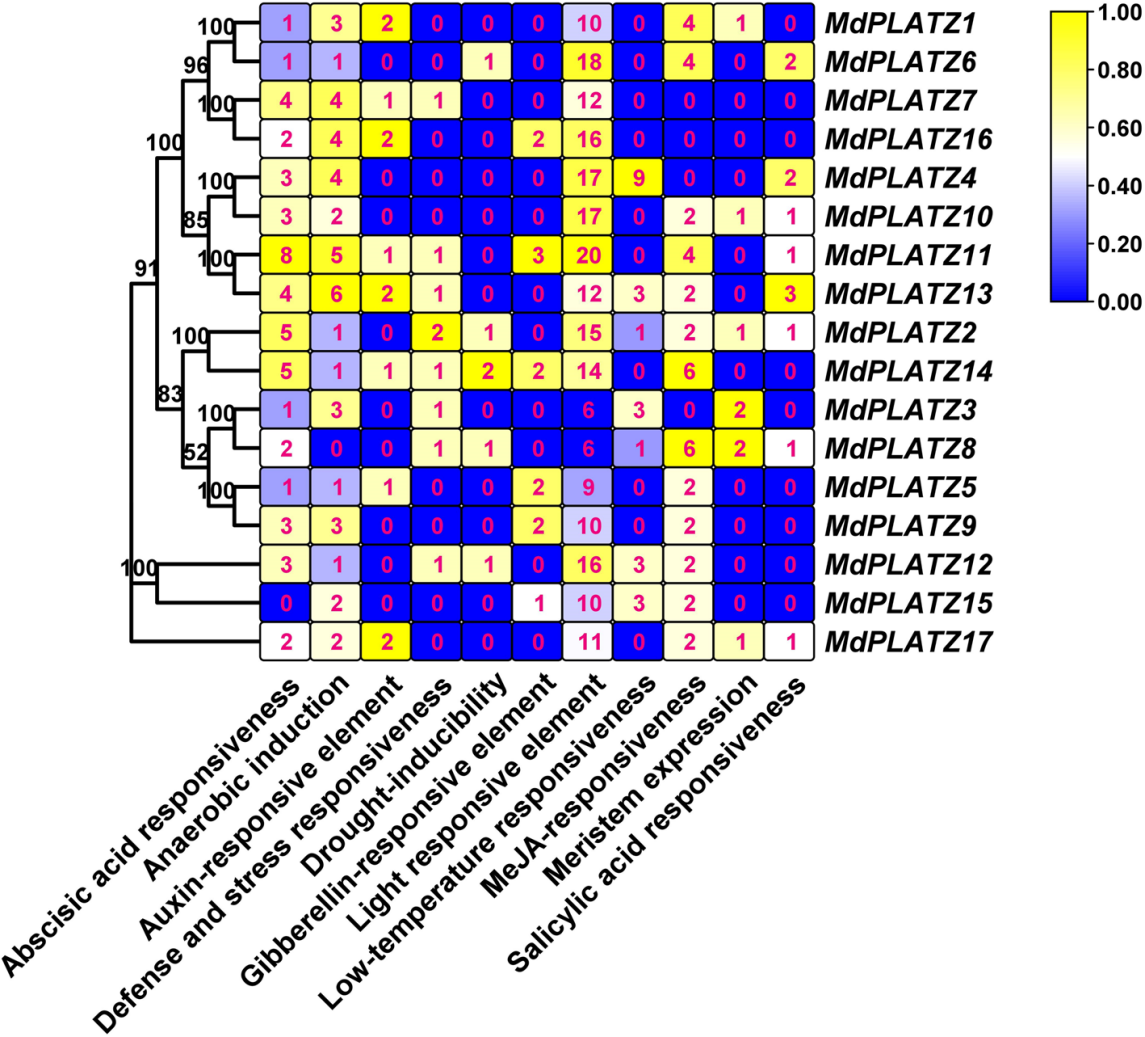
Analysis of *cis*-acting elements in the promoter of *MdPLATZ* genes. The color scale represents log_2_ transformed number of *cis*-acting elements. Yellow represents a high number of *cis*-acting elements. Blue represents a small number of *cis*-acting elements. The numbers in the colored boxes are the numbers of *cis*-acting elements present. The numbers on the evolutionary tree represent the bootstrap values

The 2 kb upstream of the genomic DNA sequence of the transcription start site (TSS) of the *MdPLATZ* genes was analyzed. As shown in Fig. 5, the number of *cis*-acting elements in the *MdPLATZ* genes ranged from 17 (*MdPLATZ5*) to 43 (*MdPLATZ11*). These included 219 photoresponsive elements, 124 hormone-responsive elements [to abscisic acid (48), auxin (12), gibberellin (12), methyl jasmonate (MeJA) (40), and salicylic acid (12)], 82 stress-responsive elements (to drought, low-temperature, and anaerobic stresses), and eight meristem expression-related elements (Fig. 5). All gene promoters contained a large number of light-responsive elements. In addition, abscisic acid-, MeJA-, and anaerobic- responsive elements were predicted to be present within the *MdPLATZ* promoter regions. In particular, several genes contained one or two hormone-related elements (auxin and gibberellin) in the promoter region. Moreover, six *MdPLATZ* gene promoters contained a meristem expression related element, suggesting that these *MdPLATZ* genes may play an important role in apple growth and development.

### MicroRNA analysis of *MdPLATZ* genes

To investigate the post-transcriptional regulation of the *MdPLATZ* genes, the associated microRNAs of these genes were predicted and their stem-loops were visualized. As shown in Table 2, three microRNAs were predicted for each of *MdPLATZ5*, *MdPLATZ9*, *MdPLATZ12*, and *MdPLATZ15*. Two microRNAs were predicted for each of *MdPLATZ1* and *MdPLATZ10*. A single microRNA was predicted for *MdPLATZ3*.

**Table 2:**
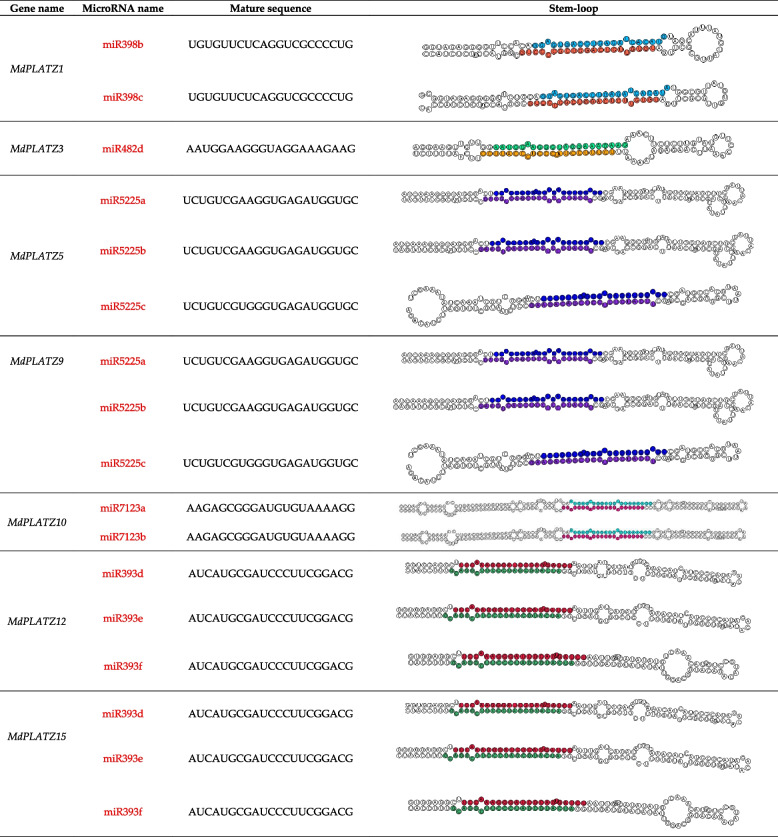
Information on the related microRNAs predicted for *MdPLATZ* genes

### Expression patterns of *MdPLATZ* genes in different tissues and at different developmental stages

**Fig. 6:**
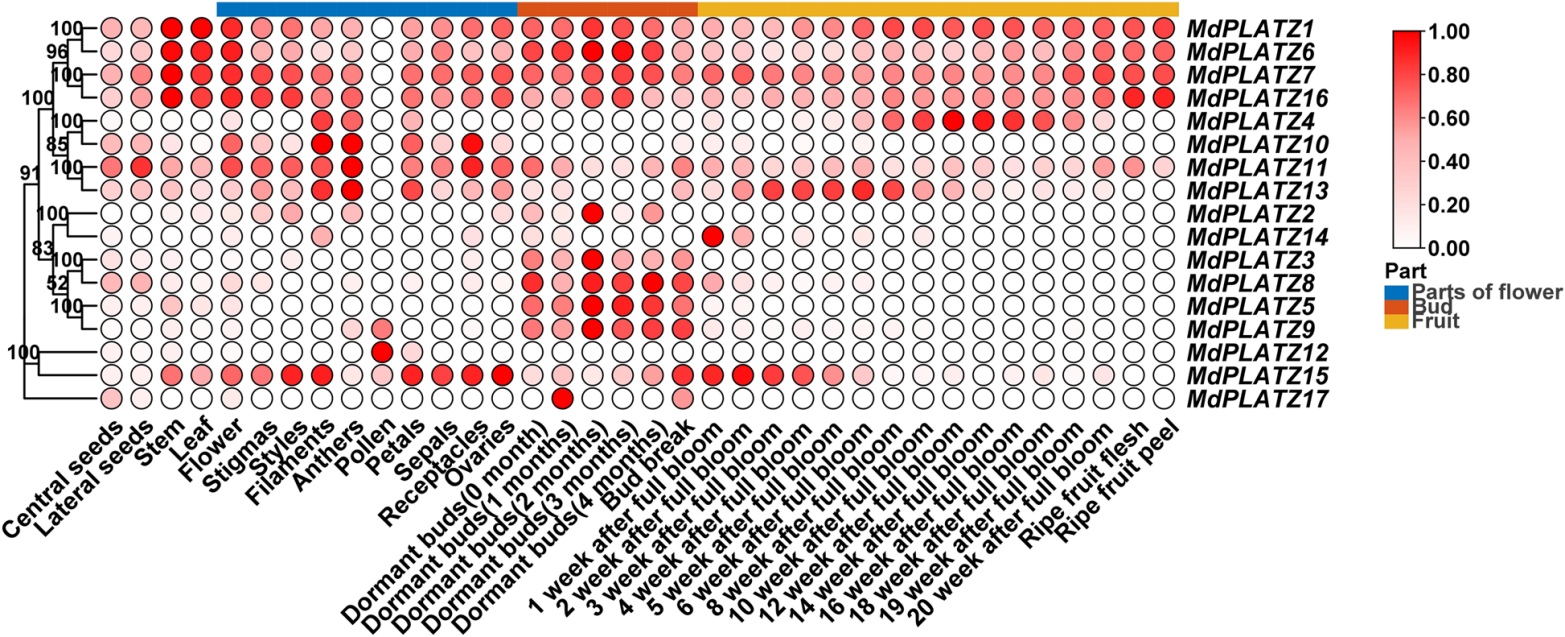
Hierarchical clustering of the expression level of apple *MdPLATZ* genes in different tissues and at different developmental stages. Transcriptome data were used to estimate the relative expression level of each gene. The heatmap shows expression patterns of *MdPLATZ* genes at different developmental stages and in different tissues. The color scale represents log_2_ transformed FPKM values; FPKM: a fragments per kilobase of transcript per million mapped reads. Deep red indicates a high expression level and light red indicates a low expression level. The numbers on the evolutionary tree represent the bootstrap values

The expression patterns of *MdPLATZ* genes in different tissues and at different developmental stages were determined and analyzed based on the apple multidimensional omics (Apple MDO) database [30]. The *MdPLATZ* genes were considered to show no expression with FPKM value less than 1. As shown in Fig. 6, *MdPLATZ1*, *MdPLATZ6*, *MdPLATZ7*, and *MdPLATZ16* were expressed in all tissues analyzed except pollen. *MdPLATZ3*, *MdPLATZ5*, *MdPLATZ8*, and *MdPLATZ9* were highly expressed in buds. *MdPLATZ2* was barely expressed in all tissues*, MdPLATZ12* was specifically expressed in pollen, *MdPLATZ14* was specifically expressed in fruit (1 week after flowering), *MdPLATZ17* was specifically expressed in dormant buds (1 month). The expression level of *MdPLATZ15* decreased gradually during fruit ripening.

### Expression patterns of *MdPLATZ* genes in response to decapitation and TDZ treatment

**Fig. 7:**
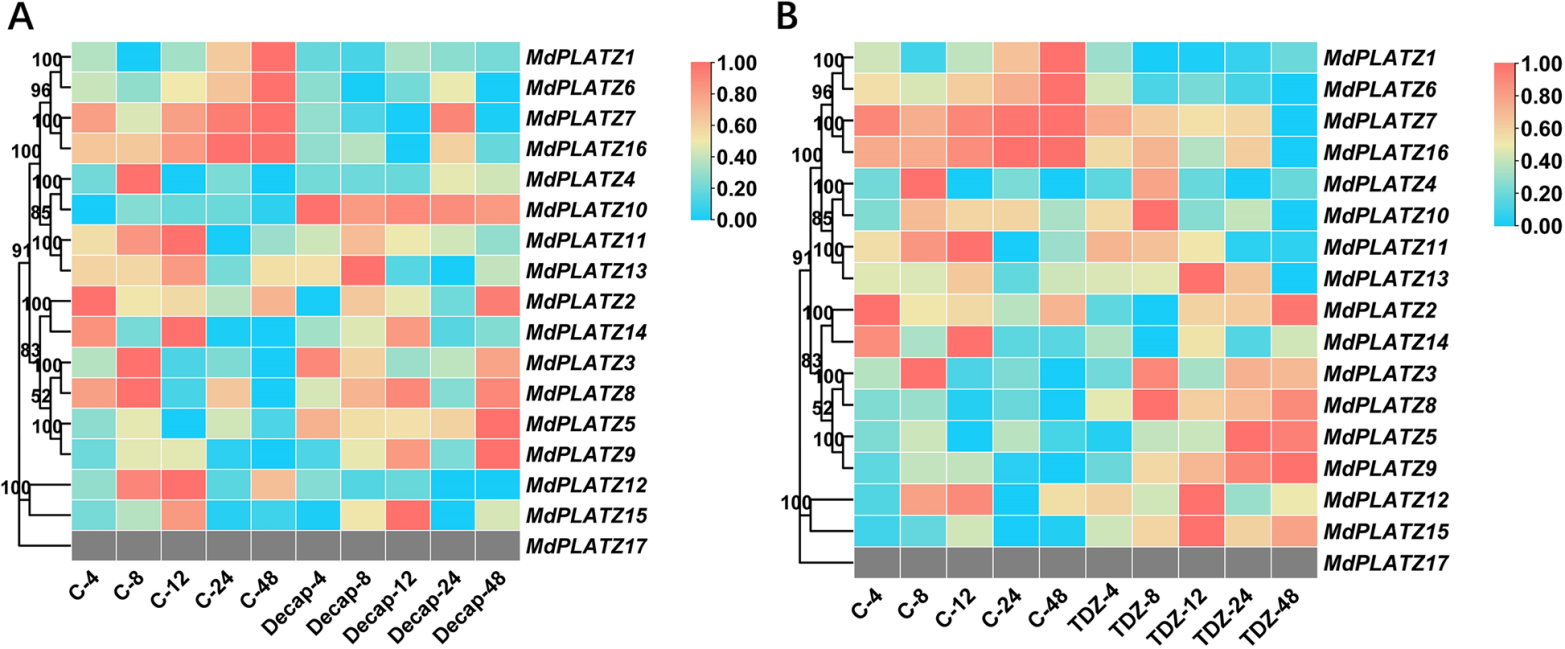
Hierarchical clustering of the expression profiles of *MdPLATZ* genes in transcriptome data for apple axillary buds after decapitation (**A**) and TDZ (**B**) treatments. “C-” represents the untreated control; “Decap-” represents the decapitation treatment. “4, 8, 12, 24, and 48” indicate the number of hours after treatment. The color scale represents log_2_ transformed FPKM values. Red indicates a high expression level and blue indicates a low expression level; grey represents no expression detected. The numbers on the evolutionary tree represent the bootstrap values

**Fig. 8:**
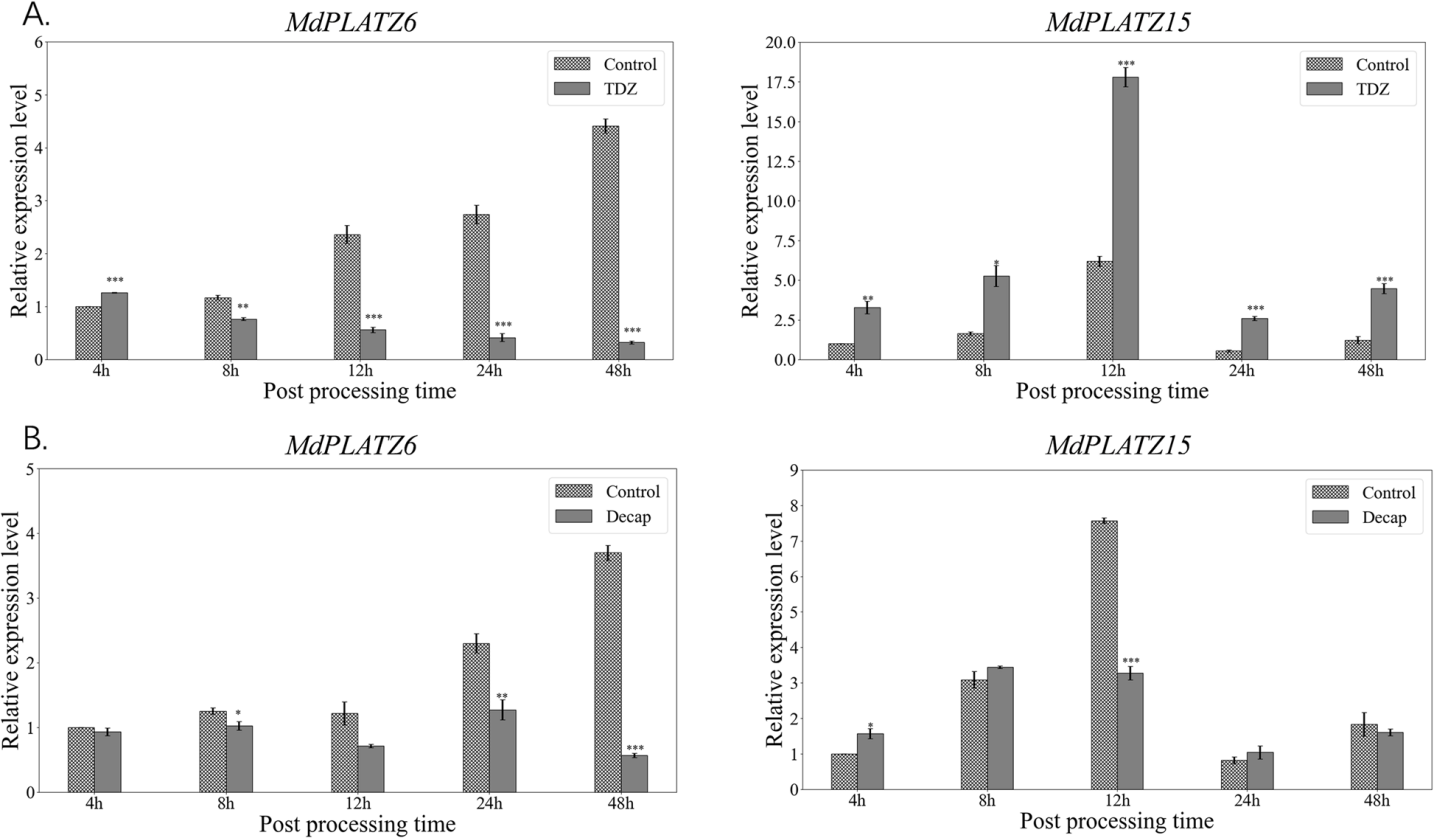
Expression patterns of *MdPLATZ6* (**A**) and *MdPLATZ15* (**B**) in axillary buds after TDZ and decapitation treatment. The relative expression levels of *MdPLATZ6* and *MdPLATZ15* were determined by qRT-PCR analysis. Error bars represent the SD of three biological replicates. Data are the mean (± SD), *n* = 3. * *P* < 0.05; ** *P* < 0.01, *** *P* < 0.001

To evaluate the putative roles of *MdPLATZ* genes in apple shoot branching, RNA-sequencing (RNA-seq) data obtained from the axillary buds after decapitation and TDZ treatment were analyzed. The expression of *MdPLATZ1*, *MdPLATZ6*, *MdPLATZ7*, and *MdPLATZ16* was downregulated in response to decapitation (Fig. 7A) and TDZ treatment (Fig. 7B), whereas *MdPLATZ8*, *MdPLATZ9*, and *MdPLATZ15* were upregulated at several times points in response to TDZ treatment (Fig. 7B). To further explore the regulation of the *MdPLATZ* genes in axillary bud outgrowth, qRT-PCR analysis was used to examine the expression of *MdPLATZ6* and *MdPLATZ15* in response to TDZ and decapitation treatments. The expression of *MdPLATZ6* was strongly downregulated in response to TDZ and decapitation at 12, 24, and 48 h, respectively (Fig. 8A). In contrast, the expression of *MdPLATZ15* was significantly upregulated in response to TDZ treatment at 4 h to 48 h; however, *MdPLATZ15* was significantly upregulated only at 4 h after decapitation (Fig. 8B).

### Interaction network analysis of MdPLATZ proteins

**Fig. 9:**
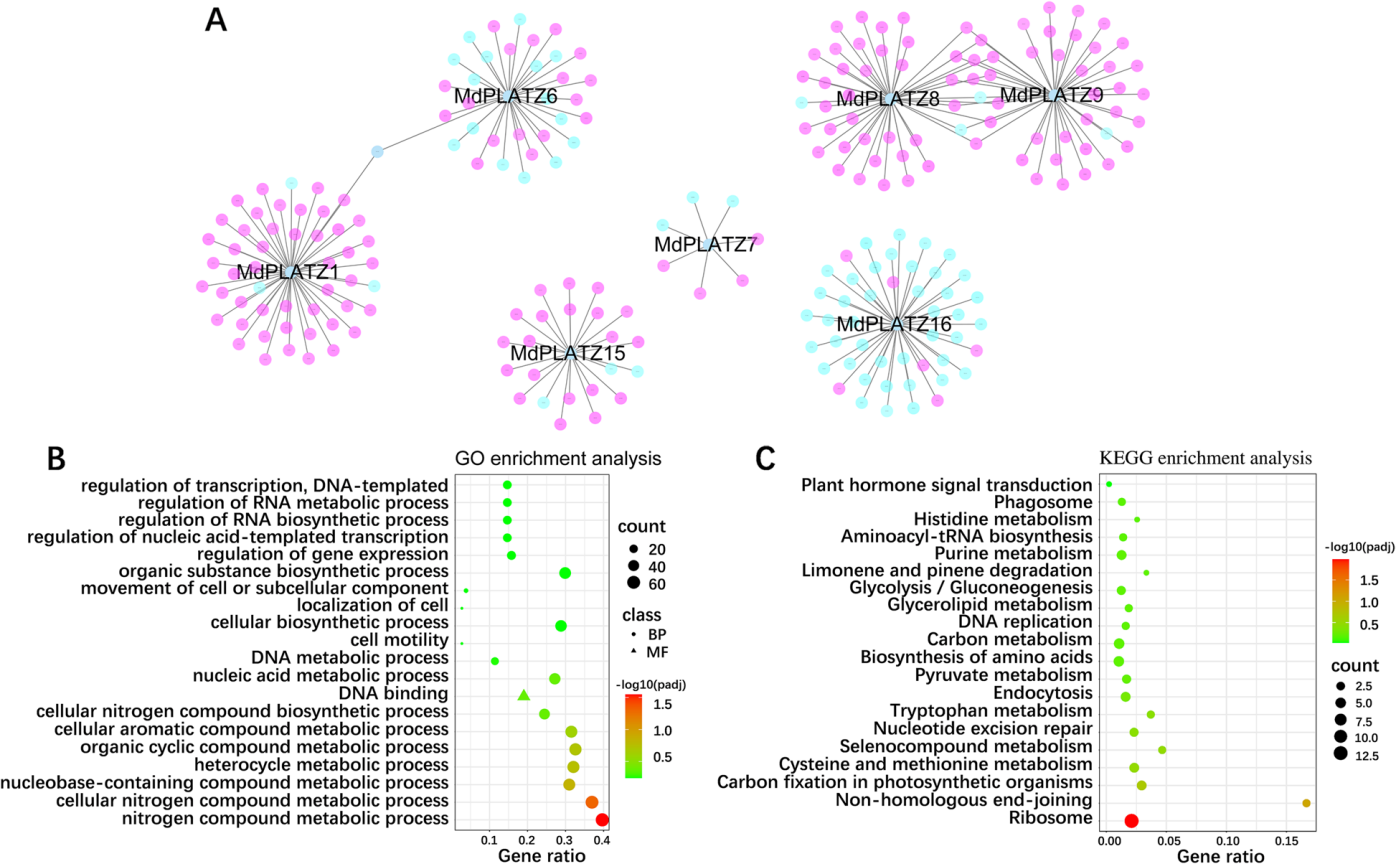
Co-expression network of MdPLATZ genes in apple. **A** Protein–protein interaction networks of seven MdPLATZ proteins that may be associated with axillary bud growth. Pink represents positive interaction, blue represents negative interaction. **B**, **C** GO and KEGG pathway enrichment analysis of MdPLATZ proteins and their co-expressed proteins. “count” represents the number of genes contained in the pathway. “class” represents the classification of pathways. BP: Biological process; MF: Molecular function. Color scale represents -log_10_ transformed corrected p-value

Protein–protein interaction (PPI) of seven MdPLATZ proteins (MdPLATZ1, MdPLATZ6, MdPLATZ7, MdPLATZ8, MdPLATZ9, MdPLATZ15, and MdPLATZ16) that may be associated with bud outgrowth was predicted and analyzed. The seven MdPLATZs were predicted to interact with 234 co-expressed proteins, including members of the bZIP, MYB, TCP, and NAC TF families (Fig. 9A, Supplementary Table S5). Additionally, MdPLATZ8 and MdPLATZ9 were significantly correlated with GRF genes (Supplementary Table S5). Gene ontology (GO) showed that the co-expressed proteins contained 20 GO categories, which belonged to cellular component, biological process and molecular function (Fig. 9B). In addition, Kyoto Encyclopedia of Genes and Genomes (KEGG) pathway enrichment analysis indicated that many co-expressed proteins were enriched into the plant hormone signal transduction, the photosynthetic carbon metabolism, and sugar metabolism pathways (Fig. 9C).

## Discussion

In recent years, genome-wide analysis of the *PLATZ* gene family in numerous plant species has been conducted. The genes perform different functions in plant growth and development, and response to stress. In this study, *17 MdPLATZ* family members in apple were identified, which is more than identified in watermelon (10), *Arabidopsis* (12), and rice (15). Thus, the *PLATZ* family members have expanded in the apple genome during evolution. The identification and chromosome locations of 17 *MdPLATZ* genes were consistent with Sun's research [7], but with a difference in naming methods of *MdPLATZ* genes. Generally, TFs are located in the cytoplasm and are transported to the nucleus, where they interact with *cis*-acting elements after receiving cell membrane signal transduction signals [31]. In this study, the predicted subcellular localization of MdPLATZ proteins was mostly in the nucleus, except for MdPLATZ5 (predicted to be localized to chloroplasts). The proteins that were localized in the nucleus had at least one signal peptide, except MdPLATZ17. This indicated that MdPLATZ17 may bind to another protein and be transported into the nucleus. To determine the relative chromosomal positions of the *MdPLATZ* genes, we mapped the distribution of the 17 *MdPLATZ* genes onto the individual apple chromosomes. The results revealed that the *MdPLATZ* genes were unevenly distributed on 14 chromosomes. According to previous studies, chromosomes 3 and 11, 5 and 10, 9 and 17, and 13 and 16 of apple are mainly derived from the common ancestor [32]. In the present study, *MdPLATZ4/MdPLATZ10*, *MdPLATZ5/MdPLATZ9*, *MdPLATZ3/MdPLATZ10*, and *MdPLATZ12/MdPLATZ15* were located on chr03/chr11, chr05/chr10, chr04/chr11, and chr13/chr16, respectively, and at almost identical positions. This finding indicates that the chromosomal distribution of the genes may reflect the evolution of the species.

Sequence comparison and phylogenetic analysis of the PLATZ proteins of apple, tomato, *Arabidopsis*, rice, and maize was conducted. The phylogenetic tree resolved into three groups, and apple PLATZs were mainly clustered with *Arabidopsis* or tomato PLATZs, suggesting that the PLATZ genes in dicotyledons had a closer evolutionary relationship. In the previous research, the phylogenetic tree based on the PLATZ protein sequences of *Malus* species, *Fragaria vesca* v4.0, *Prunus persica*, *Pyrus communis* L., *A. thaliana*, and *Oryza sativa* was constructed by the maximum likelihood method, and categorized into seven groups [7]. However, it implied that PLATZs in apple were more closely related to AtPLATZs rather than OsPLATZs [7], which was consistent with our study. Each group of genes shared similar motifs and gene structures, and may perform similar biological functions. Fifteen *MdPLATZ* genes contained two distant conserved zinc finger regions, which comprised cysteine and histidine residues. The N-terminal region of the genes resembled a double zinc finger region [4], such as C3H4 or C2H5 [33]. The N-terminus of the remaining two *MdPLATZ* genes may exhibit conserved regional diversity owing to nucleotide substitutions and minor deletions [34]. Fragment replication and tandem replication are the main factors that affect the expansion of gene families and are closely associated with genetic evolution in plant genomes [35]. Therefore, we performed a synteny analysis on the *PLATZ* gene family of apple alone, and those of apple and *Arabidopsis*. This may be associated with the duplication of the apple genome, which accounts for the increased number of gene family members [36]. There are six gene pairs that exhibit fragment duplication in apple, which was consistent with previous study in apple [7]. These genes may have the same biological functions and may prevent gene loss-of-function caused by gene mutations and deletions [37]. Additionally, a comparative syntenic map between the apple and *Arabidopsis* genomes was constructed in the present study, which was not analized in Sun’s research [7]. Syntenic relationships were observed for the 10 *Arabidopsis PLATZ* genes and 13 apple *PLATZ* genes. Using this comparative genomic analysis, the function of some *PLATZ* genes may be predicted based on their homologs.

Expression of genes is regulated by *cis*-acting elements. The *cis*-acting elements of *MdPLATZ* genes related to drought stress have been analyzed in apple [7]. In the current study, the *cis*-acting elements of *MdPLATZ*s containing a variety of elements responsive to plant hormones, such as auxin, abscisic acid, and gibberellin, and other hormone-responsive elements that were reported to associate with bud outgrowth were focused. It has been shown that PLATZ TFs play a role in regulating cell proliferation and division [38], indicating that they may participate in apple branch development. MicroRNAs are a type of endogenous non-coding small RNA molecules that exist widely among plants [39]. They inhibit the expression of target genes at the post-transcriptional level by binding to the target-gene mRNA [40]. In the present study, we observed that microRNA393, 398, 482, 5225, and 7123 target several *MdPLATZ* genes. Previous studies have reported that miR393 is involved in the regulation of plant taproot and root-cap growth [41], tillering, and response to biotic [42] and abiotic stresses [43]. The miR398 participates in plant oxidative stress response [44]. The miR482 increases the scavenging capacity of reactive oxygen species, promotes the accumulation of proline and other regulatory substances, and improves the salt and drought tolerance of plants. The expression of *PLATZ* genes is tissue-specific in plant. For example, pea *PLATZ* genes are specifically expressed in root tips and apical buds [3]. Cotton *PLATZ* transcripts are more abundant in the root, stem, and cotyledon, but lower in the leaf and seed [45]. *PLATZ* genes are preferentially expressed in the ear of rice at the early stages of development [10]. Similarly, in the current study, it was observed that *MdPLATZ1*, *MdPLATZ6*, *MdPLATZ7*, and *MdPLATZ16* were expressed in all organs analyzed except pollen. These genes may be important for maintaining the growth and development of apple. The expression level of *MdPLATZ15* gradually decreased with fruit ripening, which may be associated with fruit hardness. The *MdPLATZ3*, *MdPLATZ5*, *MdPLATZ8*, and *MdPLATZ9* genes were highly expressed in the bud.

The PLATZ gene family has been identified in Rosaceae species including apple, and provided evidences that PLATZ genes are involved in drought stress [7], which was consistent with previous studies [46–48]. However, in this study, we focused on the functional research of *MdPLATZ* genes in the control of plant growth and development, especially for axillary bud outgrowth, which has rarely been reported. It has been reported that the removal of plant apical buds can reduce the synthesis of auxin, thus reducing the strength of apical dominance and promoting the outgrowth of lateral buds [49]. TDZ has strong CK activity and can directly promote the outgrowth of plant axillary buds [50]. In apple, decapitation and exogenous CK treatment can significantly induce axillary bud outgrowth [51]. Hence, screening and analysis of the expression of *MdPLATZ* family members in transcriptome data for axillary buds after decapitation and TDZ treatment were performed. Seven *MdPLATZ* genes (*MdPLATZ1*, *MdPLATZ6*, *MdPLATZ7*, *MdPLATZ16*, *MdPLATZ8*, *MdPLATZ9*, and *MdPLATZ15*) were identified to associated with axillary bud outgrowth. Two genes (*MdPLATZ6* and *MdPLATZ15*) were selected for qRT-PCR analysis, and the results were similar to the transcriptome data which preliminarily verified their functions in axillary bud outgrowth, laying a foundation for further in-depth research.

The MdPLATZ co-expressed genes mainly involved in drought stress were analyzed in apple [7]. In this study, the protein co-expression network was predicted using the selected seven MdPLATZ proteins according to Fig. 7, and their co-expressing genes which were reported to associated with axillary bud outgrowth were concerned. These include bZIP, MYB, NAC, and TCP TFs. According to previous studies, *Chrysanthemum CmbZIP1* is mainly expressed in apical and axillary buds, and the number of branches in transgenic *Arabidopsis* overexpressing *CmbZIP1* is decreased compared with that of the wild type [52]. In addition, *ELONGATED HYPOCOTYL 5* of the bZIP TF family in *Arabidopsis* can significantly increase the number of branches [53]. In rice, *MYB* transcription factors are involved in the regulation of tillering [54], among which the *RAX* gene encodes R2R3-MYB TFs, which are involved in the regulation of the growth of tillering buds [55]. The tomato *Blind* gene, which is homologous to *RAX*, also affects the number of lateral buds [56]. In *Arabidopsis*, three *CUP-SHAPED COTYLEDON* genes encode NAC TFs. These three TFs affect the formation of lateral buds in *Arabidopsis* through functional redundancy [57, 58]. *BRANCHED1* (*BRC1*)/*TEOSINTE BRANCHED 1* (*TB1*), a member of class II TB1 CYCLOIDEA PCF (TCP) type TFs is considered to be a repressor of branching [59]. MdPLATZ1, down-regulated in response to decapitation and TDZ treatment (Fig. 7), was predicted to positively interact with BRC1, indicating that the role of MdPLATZ1 in axillary bud outgrowth is worth further investigation. MdPLATZ8 and MdPLATZ9 were significantly correlated with GRF genes, which were observed to modulates plant architecture [60]. Our previous study showed that apple bud outgrowth is correlated with the expression of cytokinin biosynthetic genes (isopentenyl transferase, IPT) [25]. MdPLATZ6 was predicted to negatively interact with Isopentenyl Transferase 9 (IPT9), which was belong to cytokinin biosynthetic genes and was associated with apple bud outgrowth. Additionally, a number of MdPLATZ8 and MdPLATZ9 co-expressed genes were involved in auxin pathway, such as PIN6, PIN1, ARF2, and YUCCA4, suggesting that MdPLATZ8 and MdPLATZ9 may mediate auxin to regulate axillary bud outgrowth.

## Conclusions

This study provides identification and bioinformatic analysis of the *MdPLATZ* gene family in apple. Based on the expression level in the transcriptome of axillary buds, two genes, *MdPLATZ6* and *MdPLATZ15*, were selected that may be associated with axillary bud outgrowth in apple. Through co-expression network analysis, it was demonstrated that multiple branching-related genes, including BRC1, GRF, and MYB, might be regulated by MdPLATZs. Moreover, enrichment analysis showed that *MdPLATZ* genes may regulate plant axillary bud outgrowth through CK or auxin pathway. The results lay a foundation for further research on the functions and mechanisms of *MdPLATZ* family members in axillary bud outgrowth.

## Materials and methods

### Identification and characterizations of *MdPLATZ* genes in apple

All apple protein sequences were downloaded from the Genome Database for Rosaceae (GDR; https://www.rosaceae.org/species/malus/malus_x_domestica/genome_GDDH13_v1.1). The PLATZ domain (Pfam:PF04640.17) was downloaded from the Pfam database (http://pfam-legacy.xfam.org/) to filter the putative apple PLATZ protein sequences using TBtools. All of the obtained putative apple PLATZ amino acid sequences were submitted to the Conserved Domain Database of the NCBI (http://www.ncbi.nlm.nih.gov/Structure/cdd/wrpsb.cgi) to identify the presence of domain signatures. The 17 putative MdPLATZ genes were designated *MdPLATZ1* to *MdPLATZ17* according to their chromosomal locations.

Characterization of the encoded amino acids (aa), theoretical isoelectric point (pI), molecular weight (MW), instability index, and grand average hydropathicity of the apple *MdPLATZ* genes were calculated using Expasy (https://web.expasy.org/protparam/). The subcellular localization of the proteins was predicted with Plant-mPLoc (http://www.csbio.sjtu.edu.cn/bioinf/plant-multi/#). Nuclear signal peptides were predicted using INSP (http://www.csbio.sjtu.edu.cn/bioinf/INSP/). The secondary and tertiary structures of the PLATZ proteins were predicted using SOPMA (https://npsa-prabi.ibcp.fr/cgi-bin/npsa_automat.pl?page=npsa_sopma.html) and Phyre2 (http://www.sbg.bio.ic.ac.uk/phyre2/html/page.cgi?id=index), respectively.

### Sequence alignment, phylogenetic relationships and gene structure analysis

The amino acid sequences of the MdPLATZ proteins were aligned using DNAMAN software. The sequence logos were obtained with the WebLogo tool (https://weblogo.threeplusone.com/create.cgi). The conserved domain sequences of PLATZ proteins of apple, *Arabidopsis*, tomato, rice, and maize were aligned using ClustalW. A phylogenetic tree was constructed in MEGA7 using the Neighbor-Joining (NJ) method with 1000 bootstrap replicates, and visualized and adjusted with ITOL (https://itol.embl.de/).

The conserved motifs of *MdPLATZ* genes were identified with the MEME suite (http://meme-suite.org/), and visualized with TBtools software. Gene structure (introns and exons) analysis of the *MdPLATZ* genes was conducted with TBtools.

### Chromosomal location and synteny analysis of the *MdPLATZ* genes

Information on the physical location of the *MdPLATZ* genes was obtained from the apple genome annotation gff3 format file and visualized with TBtools, which detected and visualized tandem duplication events.

The syntenic map was constructed using MCScanX and was visualized with TBtools. The non-synonymous (*K*_*a*_) and synonymous (*K*_*s*_) substitution rates of the collinear gene pairs were estimated using TBtools to predict the divergence time (*t*) and evolutionary rate (*K*_*a*_*/K*_*s*_ ratio).

### Analysis of *cis*-acting elements in the *MdPLATZ* genes

The 2,000 bp sequence upstream from the 5’-end of the *MdPLATZ* genes were extracted as the promoter region and used for *cis*-acting elements analysis with the PlantCare database (http://bioinformatics.psb.ugent.be/webtools/plantcare/html/), and visualized with TBtools.

### Expression patterns of *MdPLATZ* genes in different tissues of apple

We obtained a total of 48 expression profiles of the 17 *MdPLATZ* genes from the Apple Multi-Dimensional Omics Database (http://bioinformatics.cau.edu.cn/AppleMDO/). These profiles included 36 tissues and different developmental stages [central seed, lateral seed, stem, leaf, flower, petal, stigma, style, ovary, anther, filament, sepal, receptacle, pollen, four dormant bud stages, break bud, 14 fruit developmental stages from 1 week after full-bloom (WAF1) to harvest (WAF20), ripe fruit skin, and fruit flesh]. In this database, all RNA-seq data had been quality controlled and the FPKM values were extracted. Expression heatmaps were generated with TBtools.

### Prediction of microRNAs associated with *MdPLATZ* and interaction network analysis

The microRNAs associated with the *MdPLATZ* genes were predicted using psRNAtarget (https://www.zhaolab.org/psRNATarget/). The query Stem-loop and mature sequences were identified in the miRBase database (https://www.mirbase.org/) and plotted with sRNAminer software.

The interaction network for MdPLATZ family members was predicted using the Apple Multi-Dimensional Omics Database (http://bioinformatics.cau.edu.cn/AppleMDO/) and adjusted using Cytoscape software. The GO enrichment analysis, using the GOseq method, and KEGG pathway [61–63] enrichment analysis were conducted using KOBAS 2.0.

### Plant materials and treatments

One-year-old apple SH40 scions grafted onto *Malus robusta* Rehd. rootstocks were grown at the experimental nursery in Xiaochen village, Xiaochen Township, Li County, Hebei Province, China. The plants were cultivated in the nursery using a spacing of 50 cm × 25 cm. The following steps were used in the propagation and cultivation of the apple trees. First, seeds of *M. robusta* Rehd. were sown in spring, 2020 and then SH40 buds were grafted onto the rootstocks at approximately 20 cm above ground level in autumn, 2020. Second, the rootstock portion located above the SH40 bud was excised in spring, 2021; after pruning, only the SH40 buds were allowed to grow during the growing period. The one-year-old SH40/*M. robusta* Rehd. grafted combinations were used in the experiments in July, 2021. The plant materials were subjected to standard management practices.

The decapitation and exogenous TDZ treatments were executed on the grafted apple trees when the terminal shoot had attained 70–80 cm in length (during July, 2021), as measured from the grafting point to the tip of the main shoot. Eight full axillary buds were marked continuously in the apical sections of newly developing scion shoots. Exogenous TDZ solution (5 mmol/L) supplemented with 0.05% DIMETHYL SULFOXIDE (DMSO) was applied to the marked section of the scion (containing the eight axillary buds) as a single spray application. The solution was applied by hand using a 500 mL Solo Snazzy pressurized hand sprayer. For the decapitation treatment, the approximately 2 cm apical portion of the terminal shoot was decapitated. Each treatment comprised three biological replicates, with 15 plants per replicate. Axillary buds were sampled at 4, 8, 12, 24, and 48 h after treatment. All sampled materials were immediately frozen in liquid nitrogen and stored at − 80 °C until further use. The transcriptome was sequenced by Tianjin Nuohe Zhiyuan Bioinformation Technology Co., Ltd.

### Total RNA extraction and qRT-PCR analysis

Total RNA was extracted using the RNA Plant Plus Reagent Kit (Tiangen, Beijing, China) in accordance with the manufacturer’s instructions. The cDNA was synthesized with the EasyScript® One-Step gDNA Removal and cDNA Synthesis SuperMix (TransGen, Beijing, China) following the manufacturer’s instructions. The qRT-PCR assays were performed with the SYBR Green PCR Master Mix on a LightCycler® 96 system (Roche, Basel, Switzerland). All reactions included 12.5 μL of 2 × SYBR Premix Ex Taq II (Accurate Biotechnology, Hunan, China), 1.0 μL cDNA template, 0.5 μL forward and reverse primers, and 10.5 μL ddH_2_O, made up to a 25 μL volume. The thermal-cycling protocol consisted of 95 °C for 30 s, followed by 40 cycles of 95 °C for 5 s and 60 °C for 30 s, followed by melting curve analysis. Quantitative estimation of gene expression was calculated using the 2^−ΔΔCt^ method [64]. The *MdACTIN* gene served as a reference gene. All primers used in the qRT-PCR analyses were designed using Primer Premier 6.0 software. Primer information presented in Supplementary Table S1.

### Statistical analysis

Statistical analysis of qRT-PCR data was performed in Microsoft Excel. Significant differences in the data were analyzed using the Statistical Program for Social Science 18 (SPSS, Chicago, IL, USA). Differences in values were considered statistically significant at (* *P* < 0.05; ** *P* < 0.01, *** *P* < 0.001) level according to independent samples t-test. Figures were generated by Python.

## Supplementary Information

**Additional file 1: Supplementary Table S1.** Primers used for qRT-PCR. **Supplementary Table S2.** Information of the secondary protein structures of apple MdPLATZs. **Supplementary Table S3.** Collinear gene pairs of the apple MdPLATZ gene family. **Supplementary Table S4.** Ka and Ks analysis of PLATZ genes in apple and Arabidopsis. **Supplementary Table S5.** The candidate genes co-expressed with MdPLATZs in apple. **Fig. S1.** Predicted tertiary protein structures of MdPLATZ proteins. Percentages represent credibility. **Fig. S2.** Multiple sequence alignments of MdPLATZ proteins. 

## Data Availability

All data generated during this study are included in this published article [and its supplementary information files].
