# Supplementary material for: Genome-wide analysis of MdPLATZ genes and their expression during axillary bud outgrowth in apple (Malus domestica Borkh.)
Source: BMC Genomics. 2023 Jun 15;24:329. doi: 10.1186/s12864-023-09399-x (PMC10268484; doi:10.1186/s12864-023-09399-x)
Supplement: Supplementary file 1 — Additional file 1: Supplementary Table S1. Primers used for qRT-PCR. Supplementary Table S2. Information of the secondary protein structures of apple MdPLATZs. Supplementary Table S3. Collinear gene pairs of the apple MdPLATZ gene family. Supplementary Table S4. Ka and Ks analysis of PLATZ genes in apple and Arabidopsis. Supplementary Table S5. The candidate genes co-expressed with MdPLATZs in apple. Fig. S1. Predicted tertiary protein structures of MdPLATZ proteins. Percentages represent credibility. Fig. S2. Multiple sequence alignments of MdPLATZ proteins. [file 12864_2023_9399_MOESM1_ESM.docx]

Genome-wide analysis of *MdPLATZ* genes and their expression during axillary bud outgrowth in apple (*Malus domestica* Borkh.)

Jiuyang Li, Yongliang Zhao, Yaohui Zhang, Feng Ye, Zhengcun Hou, Yuhang Zhang, Longjie Hao, Guofang Li, Jianzhu Shao* and Ming Tan*

College of Horticulture, Hebei Agricultural University, Baoding 071000, Hebei, China

∗ Correspondence: Jianzhu Shao yysjz@hebau.edu.cn; Ming Tan tanming@hebau.edu.cn

**Supplementary Table S1-5**

**Supplementary Fig. S1-2**

**Supplementary Table S1. Primers used for qRT-PCR.**

| **Primer name** | **Sequences (5' to 3')** |
| --- | --- |
| *Actin-F* | TGACCGAATGAGCAAGGAAATTACT |
| *Actin-R* | TACTCAGCTTTGGCAATCCACATC |
| *MdPLATZ6-F* | AGCACGGCAGGGATGGTTTCTA |
| *MdPLATZ6-R* | GTCTTCTGGCATTGTGGTGGTTGT |
| *MdPLATZ15-F* | TGACGCCGAAAGACCACCAAAG |
| *MdPLATZ15-R* | CCGCTAACGAGATAGAGGACGACT |

**Supplementary Table S2 Information of the secondary protein structures of apple MdPLATZs.**

| **Gene name** | **Alpha helix (Hh)** | **Extended strand (Ee)** | **Beta turn (Tt)** | **Random coil (Cc)** |
| --- | --- | --- | --- | --- |
| *MdPLATZ1* | 0.2063 | 0.1508 | 0.0238 | 0.619 |
| *MdPLATZ2* | 0.284 | 0.1089 | 0.0272 | 0.5798 |
| *MdPLATZ3* | 0.2715 | 0.1041 | 0.0226 | 0.6018 |
| *MdPLATZ4* | 0.2083 | 0.1667 | 0.0677 | 0.5573 |
| *MdPLATZ5* | 0.2205 | 0.1063 | 0.0079 | 0.6654 |
| *MdPLATZ6* | 0.1548 | 0.1429 | 0.0238 | 0.6786 |
| *MdPLATZ7* | 0.1847 | 0.1982 | 0.0315 | 0.5856 |
| *MdPLATZ8* | 0.2735 | 0.1061 | 0.0245 | 0.5959 |
| *MdPLATZ9* | 0.2118 | 0.1569 | 0.0353 | 0.5961 |
| *MdPLATZ10* | 0.1698 | 0.1698 | 0.0425 | 0.6179 |
| *MdPLATZ11* | 0.2335 | 0.1827 | 0.0305 | 0.5533 |
| *MdPLATZ12* | 0.3271 | 0.1589 | 0.0467 | 0.4673 |
| *MdPLATZ13* | 0.2183 | 0.1827 | 0.0305 | 0.5685 |
| *MdPLATZ14* | 0.2314 | 0.1451 | 0.0275 | 0.5961 |
| *MdPLATZ15* | 0.2775 | 0.1366 | 0.0352 | 0.5507 |
| *MdPLATZ16* | 0.177 | 0.1726 | 0.031 | 0.6195 |
| *MdPLATZ17* | 0.2857 | 0.1701 | 0.0476 | 0.4966 |

**Supplementary Table S3 Collinear gene pairs of the apple *MdPLATZ* gene family.**

| **Duplicate 1** | **Gene ID** | **Duplicate 2** | **Gene ID** |
| --- | --- | --- | --- |
| *MdPLATZ3* | *MD02G1208800* | *MdPLATZ8* | *MD07G1117000* |
| *MdPLATZ2* | *MD02G1017000* | *MdPLATZ14* | *MD15G1161500* |
| *MdPLATZ4* | *MD03G1129200* | *MdPLATZ10* | *MD11G1151300* |
| *MdPLATZ5* | *MD05G1248500* | *MdPLATZ9* | *MD10G1229000* |
| *MdPLATZ7* | *MD06G1035500* | *MdPLATZ16* | *MD16G1273400* |
| *MdPLATZ12* | *MD13G1017800* | *MdPLATZ15* | *MD16G1015800* |

**Supplementary Table S4 *K_a_* and *K_s_* analysis of *PLATZ* genes in apple and *Arabidopsis*.**

| **Seq_1** | **Seq_2** | ***K_a_*^a^** | ***K*_s_^b^** | ***K_a_*/*K_s_*** |
| --- | --- | --- | --- | --- |
| *MD02G1208800* | *MD07G1117000* | 0.030476514 | 0.170036769 | 0.18 |
| *MD02G1017000* | *MD15G1161500* | 0.029850444 | 0.134051386 | 0.22 |
| *MD03G1129200* | *MD11G1151300* | 0.066604409 | 0.158995365 | 0.42 |
| *MD05G1248500* | *MD10G1229000* | 0.038537373 | 0.150188028 | 0.26 |
| *MD06G1035500* | *MD16G1273400* | 0.019621786 | 0.242398279 | 0.08 |
| *MD13G1017800* | *MD16G1015800* | 0.251745411 | 0.417172313 | 0.60 |
| *AT2G12646.1* | *MD02G1017000* | 0.254714694 | 1.963880268 | 0.13 |
| *AT3G60670.1* | *MD02G1208800* | 0.353176511 | 2.047013591 | 0.17 |
| *AT2G27930.6* | *MD03G1129200* | 0.36484296 | 1.884093619 | 0.19 |
| *AT1G31040.1* | *MD05G1248500* | 0.240743375 | 2.638254279 | 0.09 |
| *AT1G21000.1* | *MD06G1001300* | 0.189162106 | NaN^c^ | NaN |
| *AT1G76590.1* | *MD06G1001300* | 0.217484877 | 2.698497718 | 0.08 |
| *AT1G32700.1* | *MD06G1035500* | 0.195670201 | 1.998865676 | 0.10 |
| *AT4G17900.1* | *MD06G1035500* | 0.174261065 | 1.380139547 | 0.13 |
| *AT5G46710.1* | *MD06G1035500* | 0.305639216 | NaN | NaN |
| *AT3G60670.1* | *MD07G1117000* | 0.306813191 | 3.502404875 | 0.09 |
| *AT1G31040.1* | *MD10G1229000* | 0.225957359 | 3.098470424 | 0.07 |
| *AT2G27930.6* | *MD11G1151300* | 0.349197712 | 1.673305472 | 0.21 |
| *AT2G01818.1* | *MD13G1017800* | 0.468378057 | 2.787787748 | 0.17 |
| *AT2G12646.1* | *MD15G1161500* | 0.243792244 | 1.755411551 | 0.14 |
| *AT2G01818.1* | *MD16G1015800* | 0.38471729 | NaN | NaN |
| *AT1G32700.1* | *MD16G1273400* | 0.164555576 | NaN | NaN |
| *AT5G46710.1* | *MD16G1273400* | 0.324741417 | NaN | NaN |

^a^ *K_a_*: number of SNPs with non-synonymous substitution/number of non-synonymous substitution bits.

^b^ *K_s_*: number of SNPs with synonymous replacement/number of synonymous replacement bits.

^c^ NaN means that it cannot be calculated.

| **Supplementary Table S5 The candidate genes co-expressed with MdPLATZs in apple** | | | | |
| --- | --- | --- | --- | --- |
| **Source_id** | **Target_id** | **gene accession** | **Annotation** | **Relationship** |
| MdPLATZ1 | MD12G1241100 | BZIP63 | bZIP transcription factor family protein | positive |
| MdPLATZ1 | MD16G1057900 | AT1G13530.1 | Protein of unknown function (DUF1262) | positive |
| MdPLATZ1 | MD17G1158100 | AT3G13062.1 | Polyketide cyclase/dehydrase and lipid transport superfamily protein | positive |
| MdPLATZ1 | MD16G1014000 | XTH28 | xyloglucan endotransglucosylase/hydrolase 28 | positive |
| MdPLATZ1 | MD04G1224400 | BZIP63 | bZIP transcription factor family protein | positive |
| MdPLATZ1 | MD08G1104300 | LRR4 | NB-ARC domain-containing disease resistance protein | positive |
| MdPLATZ1 | MD16G1129900 | CCL, CCR-LIKE | CCR-like protein | positive |
| MdPLATZ1 | MD06G1234200 | AT3G17770.1 | Dihydroxyacetone kinase | positive |
| MdPLATZ1 | MD13G1185400 |  | Plant protein of unknown function (DUF828) | positive |
| MdPLATZ1 | MD02G1103900 | FAB1B | phosphatidylinositol-4-phosphate 5-kinase family protein | positive |
| MdPLATZ1 | MD08G1102200 | PGSIP3, GUX2 | plant glycogenin-like starch initiation protein 3 | positive |
| MdPLATZ1 | MD10G1074000 | BH063 | cryptochrome-interacting basic-helix-loop-helix 1 | positive |
| MdPLATZ1 | MD09G1008300 | BRC1 | TEOSINTE BRANCHED 1 | positive |
| MdPLATZ1 | MD13G1079300 | PTR2 | nitrate transporter 1:2 | positive |
| MdPLATZ1 | MD07G1298900 | AT5G54180.1 | 30S ribosomal protein | positive |
| MdPLATZ1 | MD05G1130000 | ALATS | Alanyl-tRNA synthetase | positive |
| MdPLATZ1 | MD06G1175500 |  | Protein of unknown function (DUF3506) | positive |
| MdPLATZ1 | MD02G1259000 | CAT7 | cationic amino acid transporter 7 | positive |
| MdPLATZ1 | MD16G1179400 | AT4G15530 | pyruvate orthophosphate dikinase | positive |
| MdPLATZ1 | MD10G1179700 | AT4G28570 | Long-chain fatty alcohol dehydrogenase family protein | positive |
| MdPLATZ1 | MD10G1074400 | AT1G75250.1 | RAD-like 6 | positive |
| MdPLATZ1 | MD11G1299900 | GRXC11 | Glutaredoxin-C11 | positive |
| MdPLATZ1 | MD04G1183400 | DEGP7 | DEAD box RNA helicase (RH3) | positive |
| MdPLATZ1 | MD06G1038500 | FTSHB | FTSH protease 11 | positive |
| MdPLATZ1 | MD15G1155500 | TIL | temperature-induced lipocalin | positive |
| MdPLATZ1 | MD09G1197500 | AT1G73040 | Mannose-binding lectin superfamily protein | positive |
| MdPLATZ1 | MD04G1028000 | P2C33 | Protein phosphatase 2C family protein | positive |
| MdPLATZ1 | MD08G1104200 | LRR4 | NB-ARC domain-containing disease resistance protein | positive |
| MdPLATZ1 | MD06G1188000 |  | Uncharacterised conserved protein UCP015417 | positive |
| MdPLATZ1 | MD15G1445500 |  | Protein of unknown function | positive |
| MdPLATZ1 | MD15G1183500 | PP395 | protein kinase family protein | positive |
| MdPLATZ1 | MD11G1197100 | DER | GTP-binding family protein | positive |
| MdPLATZ1 | MD05G1130100 | ALATS | Alanyl-tRNA synthetase | positive |
| MdPLATZ1 | MD00G1061700 | AMPD | AMP deaminase | positive |
| MdPLATZ1 | MD15G1217500 |  | Protein of unknown function | positive |
| MdPLATZ1 | MD16G1064200 | AT2G03440.1 | nodulin-related protein 1 | positive |
| MdPLATZ1 | MD04G1247400 | HCF109 | high chlorophyll fluorescent 109 | positive |
| MdPLATZ1 | MD09G1194600 | AT4G33000 | calcineurin B-like protein 10 | positive |
| MdPLATZ1 | MD04G1128000 | CNGC1 | cyclic nucleotide gated channel 1 | positive |
| MdPLATZ1 | MD15G1075500 | C81D1 | cytochrome P450 | positive |
| MdPLATZ1 | MD06G1067800 | ESCA | Predicted AT-hook DNA-binding family protein | positive |
| MdPLATZ1 | MD06G1130300 | EF114 | Integrase-type DNA-binding superfamily protein | positive |
| MdPLATZ1 | MD04G1191200 | PPD1 | Photosystem II reaction center PsbP family protein | positive |
| MdPLATZ1 | MD02G1166700 | UIF1 | Homeodomain-like superfamily protein | positive |
| MdPLATZ1 | MD03G1209400 | ETFA | electron transfer flavoprotein alpha | positive |
| MdPLATZ1 | MD15G1149600 | AT5G57860.2 | FAD/NAD(P)-binding oxidoreductase family protein | positive |
| MdPLATZ1 | MD04G1101600 | FIM1 | fimbrin 1 | positive |
| MdPLATZ1 | MD16G1281300 | AT4G19006.2 | Proteasome component (PCI) domain protein | negative |
| MdPLATZ1 | MD02G1094700 | MTI1 | 5-methylthioribose-1-phosphate isomerase. | negative |
| MdPLATZ1 | MD06G1137700 | EL8Y | Ribosomal protein L7Ae/L30e/S12e/Gadd45 family protein | negative |
| MdPLATZ1 | MD09G1186200 | NFYA3 | nuclear factor Y | predicted PPI |
| MdPLATZ6 | MD16G1037700 |  | Protein of unknown function (DUF789) | positive |
| MdPLATZ6 | MD11G1315500 | AT1G02610.1 | RING/FYVE/PHD zinc finger superfamily protein | positive |
| MdPLATZ6 | MD08G1096000 | ERF08 | DREB and EAR motif protein 3; encodes a member of the DREB subfamily A-5 of ERF/AP2 transcription factor family. The protein contains one AP2 domain. There are 16 members in this subfamily including RAP2.1, RAP2.9 and RAP2.10. | positive |
| MdPLATZ6 | MD11G1241900 | HUP54 | Aluminium induced protein with YGL and LRDR motifs | positive |
| MdPLATZ6 | MD04G1054000 | AT4G17080.1 | Histone H3 K4-specific methyltransferase SET7/9 family protein | positive |
| MdPLATZ6 | MD10G1072800 | FDA10, PCH1 | F-box family protein | positive |
| MdPLATZ6 | MD03G1266100 | EMB2289, SCY2 | SecY protein transport family protein | positive |
| MdPLATZ6 | MD05G1033400 | GIGAN | gigantea protein (GI) | positive |
| MdPLATZ6 | MD03G1015500 | NOC2L | LRR and NB-ARC domains-containing disease resistance protein | positive |
| MdPLATZ6 | MD07G1070500 | Y5393 | Protein kinase superfamily protein | positive |
| MdPLATZ6 | MD15G1138800 |  | Plant protein of unknown function (DUF869) | positive |
| MdPLATZ6 | MD02G1151700 | MGP | C2H2-like zinc finger protein | positive |
| MdPLATZ6 | MD10G1054700 |  | Protein of unknown function (DUF3506) | positive |
| MdPLATZ6 | MD07G1310100 | -- | -- | positive |
| MdPLATZ6 | MD00G1187300 | -- | -- | positive |
| MdPLATZ6 | MD01G1217700 |  | Protein of unknown function | positive |
| MdPLATZ6 | MD15G1226200 | KASC1 | 2-oxoglutarate (2OG) and Fe(II)-dependent oxygenase superfamily protein | positive |
| MdPLATZ6 | MD05G1234200 | PGK3, PGKC | phosphoglycerate kinase | negative |
| MdPLATZ6 | MD16G1033600 | AT5G08680 | ATP synthase alpha/beta family protein | negative |
| MdPLATZ6 | MD16G1078300 | VDAC1 | voltage dependent anion channel 1 | negative |
| MdPLATZ6 | MD16G1115300 | CI51; NDUFV1 | 51 kDa subunit of complex I | negative |
| MdPLATZ6 | MD08G1029100 | GAMMA CA1 | gamma carbonic anhydrase 1 | negative |
| MdPLATZ6 | MD12G1008100 | ATCIMS, METS1 | Cobalamin-independent synthase family protein | negative |
| MdPLATZ6 | MD16G1095100 | ARFA1E | ADP-ribosylation factor A1E; A member of ARF GTPase family | negative |
| MdPLATZ6 | MD07G1110200 | NDK1_PEA | Nucleoside diphosphate kinase family protein | negative |
| MdPLATZ6 | MD10G1031700 |  | Protein of unknown function | negative |
| MdPLATZ6 | MD05G1165200 | RPSAB, US2Y | 40s ribosomal protein SA | negative |
| MdPLATZ6 | MD13G1093800 | ARFA1F | ADP-ribosylation factor A1F | negative |
| MdPLATZ6 | MD13G1111500 | G3PC | glyceraldehyde-3-phosphate dehydrogenase C2 | negative |
| MdPLATZ6 | MD03G1045500 | CSN6B | COP9 signalosome subunit 6A | negative |
| MdPLATZ6 | MD15G1202600 | RL281 | Ribosomal L28e protein family | negative |
| MdPLATZ6 | MD11G1259400 | IPT9 | Lactate/malate dehydrogenase family protein IPT9 | negative |
| MdPLATZ6 | MD09G1186200 | NFYA3 | nuclear factor Y | predicted PPI |
| MdPLATZ7 | MD00G1029100 | ALDH3H1 | aldehyde dehydrogenase 3H1 | positive |
| MdPLATZ7 | MD16G1020100 | ACCH1 | 2-oxoglutarate (2OG) and Fe(II)-dependent oxygenase superfamily protein | positive |
| MdPLATZ7 | MD15G1139900 | Y5129 | Leucine-rich repeat protein kinase family protein | positive |
| MdPLATZ7 | MD10G1226500 | AT1G30910 | Molybdenum cofactor sulfurase family protein | positive |
| MdPLATZ7 | MD03G1080000 | TUB8 | tubulin beta 8 | negative |
| MdPLATZ7 | MD08G1014300 | GH9B7 | glycosyl hydrolase 9B7 | negative |
| MdPLATZ7 | MD10G1089700 | AT4G33945 | ARM repeat superfamily protein | negative |
| MdPLATZ8 | MD02G1201000 | GRF9 | growth-regulating factor 9 | positive |
| MdPLATZ8 | MD09G1011500 | FIGL1 | P-loop containing nucleoside triphosphate hydrolases superfamily protein | positive |
| MdPLATZ8 | MD08G1043600 | ATH1 | homeobox gene 1 | positive |
| MdPLATZ8 | MD06G1236400 | HDG11 | homeodomain GLABROUS 11 | positive |
| MdPLATZ8 | MD01G1198500 | EXS | Leucine-rich repeat transmembrane protein kinase | positive |
| MdPLATZ8 | MD09G1183300 | GRF5 | growth-regulating factor 5 | positive |
| MdPLATZ8 | MD11G1285300 | -- | -- | positive |
| MdPLATZ8 | MD15G1099400 |  | Protein of unknown function (DUF630 and DUF632) | positive |
| MdPLATZ8 | MD04G1227900 | AT5G28640 | SSXT family protein | positive |
| MdPLATZ8 | MD04G1177100 | AT3G08570 | Phototropic-responsive NPH3 family protein | positive |
| MdPLATZ8 | MD05G1342200 | PDF2 | protodermal factor 2 | positive |
| MdPLATZ8 | MD03G1218100 | MRE11 | DNA repair and meiosis protein (Mre11) | positive |
| MdPLATZ8 | MD10G1315800 | PDF2 | protodermal factor 2 | positive |
| MdPLATZ8 | MD15G1049300 | ERECT | Leucine-rich receptor-like protein kinase family protein | positive |
| MdPLATZ8 | MD15G1140600 |  | INVOLVED IN: biological process unknown | positive |
| MdPLATZ8 | MD04G1184400 | PP284 | 5'-3' exonuclease family protein | positive |
| MdPLATZ8 | MD17G1012100 | FIGL1 | P-loop containing nucleoside triphosphate hydrolases superfamily protein | positive |
| MdPLATZ8 | MD08G1060500 | CTC1 | conserved telomere maintenance component 1 | positive |
| MdPLATZ8 | MD07G1259500 | ERL1 | ERECTA-like 1 | positive |
| MdPLATZ8 | MD07G1283200 |  | Protein of unknown function | positive |
| MdPLATZ8 | MD16G1173400 | AT5G18590 | Galactose oxidase/kelch repeat superfamily protein | positive |
| MdPLATZ8 | MD15G1105700 | TOP1 | DNA topoisomerase | positive |
| MdPLATZ8 | MD09G1205400 | ATHB15, CAN | Homeobox-leucine zipper family protein / lipid-binding START domain-containing protein; Member of the class III HD-ZIP protein family | positive |
| MdPLATZ8 | MD15G1088300 | -- | -- | positive |
| MdPLATZ8 | MD05G1124400 |  | Protein of unknown function (DUF630 and DUF632) | positive |
| MdPLATZ8 | MD14G1137600 | GRF8 | growth-regulating factor 8 | positive |
| MdPLATZ8 | MD07G1008800 | EMB133, TTN1 | ARM repeat superfamily protein | positive |
| MdPLATZ8 | MD10G1014300 | PIN6 | Auxin efflux carrier family protein | positive |
| MdPLATZ8 | MD07G1297900 | IQD17 | IQ-domain 17 | positive |
| MdPLATZ8 | MD06G1012500 | JMJ29 | transcription factor jumonji (jmjC) domain-containing protein; Encodes a probable H3K9me2 demethylase | positive |
| MdPLATZ8 | MD02G1093600 |  | Protein of unknown function | positive |
| MdPLATZ8 | MD13G1105600 | TE1 | terminal EAR1-like 1 | positive |
| MdPLATZ8 | MD14G1182000 | HT1 | Protein kinase superfamily protein, involved in regulation of stomatal aperture in response to CO2. | positive |
| MdPLATZ8 | MD15G1064600 | ANT, CKC1 | Integrase-type DNA-binding superfamily protein;ANT is required for control of cell proliferation and encodes a putative transcriptional regulator similar to AP2. | positive |
| MdPLATZ8 | MD10G1229000 | AGL86 | AGAMOUS-like 86 | positive |
| MdPLATZ8 | MD14G1066700 | AT3G57660 | nuclear RNA polymerase A1; Encodes a subunit of RNA polymerase I | positive |
| MdPLATZ8 | MD16G1105800 | TEL1 | terminal EAR1-like 1 | positive |
| MdPLATZ8 | MD01G1046900 | AT3G49650.1 | P-loop containing nucleoside triphosphate hydrolases superfamily protein | positive |
| MdPLATZ8 | MD17G1012000 | FIGL1 | P-loop containing nucleoside triphosphate hydrolases superfamily protein | positive |
| MdPLATZ8 | MD17G1185400 | ATB15 | Homeobox-leucine zipper family protein / lipid-binding START domain-containing protein; | positive |
| MdPLATZ8 | MD09G1066200 | AT1G13820.1 | alpha/beta-Hydrolases superfamily protein | positive |
| MdPLATZ8 | MD17G1012200 | FIGL1 | P-loop containing nucleoside triphosphate hydrolases superfamily protein | positive |
| MdPLATZ8 | MD04G1215900 | GRF1 | growth-regulating factor 1 | positive |
| MdPLATZ8 | MD09G1087400 | RD23D | Rad23 UV excision repair protein family | negative |
| MdPLATZ8 | MD17G1076200 | RD23D | Rad23 UV excision repair protein family | negative |
| MdPLATZ8 | MD16G1235900 | KAB1, KV-BETA1 | potassium channel beta subunit 1 | negative |
| MdPLATZ9 | MD11G1253500 | NAC98 | NAC (No Apical Meristem) domain transcriptional regulator superfamily protein | positive |
| MdPLATZ9 | MD08G1165100 | AT1G44760.1 | Adenine nucleotide alpha hydrolases-like superfamily protein | positive |
| MdPLATZ9 | MD04G1227900 | AT5G28640 | SSXT family protein | positive |
| MdPLATZ9 | MD00G1056300 | ARF2 | auxin response factor 2 | positive |
| MdPLATZ9 | MD07G1297900 | IQD17 | IQ-domain 17 | positive |
| MdPLATZ9 | MD00G1114600 | -- | -- | positive |
| MdPLATZ9 | MD16G1027800 | ATHB17 | homeobox-leucine zipper protein 17 | positive |
| MdPLATZ9 | MD08G1030700 | ATHB33, HB33 | homeobox protein 33; Encodes a zinc finger-homeodomain transcription factor ZHD5 | positive |
| MdPLATZ9 | MD15G1300900 | GRF1 | growth-regulating factor 1 | positive |
| MdPLATZ9 | MD01G1037200 | AT4G24660 | homeobox protein 22 | positive |
| MdPLATZ9 | MD02G1189500 | GRF1 | growth-regulating factor 1 | positive |
| MdPLATZ9 | MD11G1285300 | -- | -- | positive |
| MdPLATZ9 | MD09G1183300 | GRF5 | growth-regulating factor 5 | positive |
| MdPLATZ9 | MD16G1089000 | AT1G60060 | Serine/threonine-protein kinase WNK (With No Lysine)-related | positive |
| MdPLATZ9 | MD02G1201000 | GRF9 | growth-regulating factor 9 | positive |
| MdPLATZ9 | MD15G1049300 | ERECT | Leucine-rich receptor-like protein kinase family protein | positive |
| MdPLATZ9 | MD06G1094800 |  | Protein of unknown function | positive |
| MdPLATZ9 | MD06G1014700 | GRF5 | growth-regulating factor 5 | positive |
| MdPLATZ9 | MD06G1232400 | KNATM | KNOTTED-like from Arabidopsis thaliana | positive |
| MdPLATZ9 | MD15G1064600 | ANT, CKC1 | Integrase-type DNA-binding superfamily protein;ANT is required for control of cell proliferation and encodes a putative transcriptional regulator similar to AP2. | positive |
| MdPLATZ9 | MD05G1124400 |  | Protein of unknown function (DUF630 and DUF632) | positive |
| MdPLATZ9 | MD06G1065700 | AT2G45190 | Plant-specific transcription factor YABBY family protein | positive |
| MdPLATZ9 | MD06G1175800 | PG2, PGL3 | polygalacturonase 2 | positive |
| MdPLATZ9 | MD09G1205400 | ATHB15, CAN | Homeobox-leucine zipper family protein / lipid-binding START domain-containing protein; Member of the class III HD-ZIP protein family | positive |
| MdPLATZ9 | MD14G1239200 | KNATM | KNOX meinox protein | positive |
| MdPLATZ9 | MD02G1093600 |  | Protein of unknown function | positive |
| MdPLATZ9 | MD17G1185400 | ATHB15, CAN | Homeobox-leucine zipper family protein / lipid-binding START domain-containing protein; Member of the class III HD-ZIP protein family | positive |
| MdPLATZ9 | MD02G1125100 | AT4G35900 | Basic-leucine zipper (bZIP) transcription factor family protein | positive |
| MdPLATZ9 | MD15G1117200 | YUC4, YUCCA4 | Flavin-binding monooxygenase family protein. Belongs to the YUC gene family. Encodes a predicted flavin monooxygenase. YUC4 is part of a pathway linking auxin biosynthesis and gynoecium development. | positive |
| MdPLATZ9 | MD15G1088300 | -- | -- | positive |
| MdPLATZ9 | MD07G1117000 | AT3G60670.1 | PLATZ transcription factor family protein | positive |
| MdPLATZ9 | MD10G1270700 | UGAGT | GDSL-like Lipase/Acylhydrolase superfamily protein | positive |
| MdPLATZ9 | MD13G1070400 | MYB105 | myb domain protein 105 | positive |
| MdPLATZ9 | MD12G1020800 | PDR6_NICPL | pleiotropic drug resistance 6 | positive |
| MdPLATZ9 | MD02G1027500 | MYB82 | myb domain protein 82 | positive |
| MdPLATZ9 | MD10G1237300 | AT2G35640 | Homeodomain-like superfamily protein | positive |
| MdPLATZ9 | MD15G1025900 | ZHD5, HB33 | homeobox protein 33; Encodes a zinc finger-homeodomain transcription factor ZHD5 | positive |
| MdPLATZ9 | MD12G1261100 | AS2 | Lateral organ boundaries (LOB) domain family protein | positive |
| MdPLATZ9 | MD12G1095100 | PIN1 | Auxin efflux carrier family protein | positive |
| MdPLATZ9 | MD16G1132900 | AT3G17380 | TRAF-like family protein | positive |
| MdPLATZ9 | MD05G1006500 | AT4G26670.1 | Mitochondrial import inner membrane translocase subunit Tim17/Tim22/Tim23 family protein | positive |
| MdPLATZ9 | MD17G1076200 | RD23D | Rad23 UV excision repair protein family | negative |
| MdPLATZ9 | MD08G1193600 | GRF6 | G-box regulating factor 6 | negative |
| MdPLATZ9 | MD09G1087400 | RD23D | Rad23 UV excision repair protein family | negative |
| MdPLATZ15 | MD14G1238400 | RPL36AA, EL42Z | Zinc-binding ribosomal protein family protein | positive |
| MdPLATZ15 | MD04G1216000 | -- | -- | positive |
| MdPLATZ15 | MD01G1133600 | CER10, TSC13 | 3-oxo-5-alpha-steroid 4-dehydrogenase family protein | positive |
| MdPLATZ15 | MD06G1230700 |  | Protein of unknown function (DUF1295) | positive |
| MdPLATZ15 | MD12G1243500 | US19Y | Ribosomal protein S19 family protein | positive |
| MdPLATZ15 | MD12G1028600 | EL40Z, RPL40A, UBQ2 | Ubiquitin-60S ribosomal protein L40 | positive |
| MdPLATZ15 | MD17G1047300 | -- | -- | positive |
| MdPLATZ15 | MD06G1101100 | AT5G41760 | Nucleotide-sugar transporter family protein | positive |
| MdPLATZ15 | MD11G1054800 | ES26X, RPS26E | Ribosomal protein S26e family protein | positive |
| MdPLATZ15 | MD07G1120500 | RPS13A, PFL2 | ribosomal protein S13A | positive |
| MdPLATZ15 | MD08G1176600 | EL30Z, RPL30A | Ribosomal protein L7Ae/L30e/S12e/Gadd45 family protein | positive |
| MdPLATZ15 | MD14G1051900 | AT3G12410 | Polynucleotidyl transferase, ribonuclease H-like superfamily protein | positive |
| MdPLATZ15 | MD00G1051100 |  | -- | positive |
| MdPLATZ15 | MD16G1242900 |  | -- | positive |
| MdPLATZ15 | MD03G1148100 | AT5G22430 | Pollen Ole e 1 allergen and extensin family protein;(source:Araport11) | positive |
| MdPLATZ15 | MD12G1023600 | FACE2 | farnesylated protein-converting enzyme 2 | positive |
| MdPLATZ15 | MD04G1010000 | FBW2 | F-BOX WITH WD-40 2 | positive |
| MdPLATZ15 | MD08G1024400 | AT1G35180 | TRAM, LAG1 and CLN8 (TLC) lipid-sensing domain containing protein | positive |
| MdPLATZ15 | MD07G1110100 | ELMOD_A | ELMO/CED-12 family protein | positive |
| MdPLATZ15 | MD11G1143300 | UXS6 | UDP-XYL synthase 6; Encodes a cytosolic isoform of UDP-glucuronic acid decarboxylase. | positive |
| MdPLATZ15 | MD17G1194400 |  | Protein of unknown function | negative |
| MdPLATZ15 | MD13G1064900 | PPA2 | Purple acid phosphatases superfamily protein | negative |
| MdPLATZ15 | MD16G1173800 | AT5G18610 | Protein kinase superfamily protein;Encodes a receptor-like cytoplasmic kinase that is an immediate downstream component of the chitin receptor CERK1 | negative |
| MdPLATZ16 | MD15G1074000 | AT5G43100 | Eukaryotic aspartyl protease family protein | positive |
| MdPLATZ16 | MD04G1239300 | MEE66 | F-box family protein | positive |
| MdPLATZ16 | MD05G1326200 | AT4G21450.1 | PapD-like superfamily protein | positive |
| MdPLATZ16 | MD11G1020500 | DRL27 | NB-ARC domain-containing disease resistance protein | positive |
| MdPLATZ16 | MD01G1154800 | LEW3 | UDP-Glycosyltransferase superfamily protein | positive |
| MdPLATZ16 | MD16G1091600 | ENT1 | equilibrative nucleotide transporter 1 | positive |
| MdPLATZ16 | MD16G1241800 | TBL5 | TRICHOME BIREFRINGENCE-LIKE 5 | negative |
| MdPLATZ16 | MD16G1209200 | AT3G23090 | TPX2 (targeting protein for Xklp2) protein family;Member of the microtubule regulatory protein WVD2/WDL family WDL3 stabilizes cortical microtubules | negative |
| MdPLATZ16 | MD08G1015000 |  | Plant protein of unknown function (DUF869) | negative |
| MdPLATZ16 | MD17G1265400 | AT3G58690 | Protein kinase superfamily protein | negative |
| MdPLATZ16 | MD07G1239000 | AT1G17360.1 | LOW protein: protein phosphatase 1 regulatory subunit-like protein;(source:Araport11) | negative |
| MdPLATZ16 | MD17G1268400 | RDS2 | HSP20-like chaperones superfamily protein | negative |
| MdPLATZ16 | MD15G1403200 | AT1G09640 | Translation elongation factor EF1B | negative |
| MdPLATZ16 | MD12G1093700 | AT3G57050 | cystathionine beta-lyase | negative |
| MdPLATZ16 | MD14G1094900 | RPL24A | ribosomal protein L24 | negative |
| MdPLATZ16 | MD06G1021300 | FEI1 | Leucine-rich repeat protein kinase family protein | negative |
| MdPLATZ16 | MD10G1037700 |  | Protein of unknown function | negative |
| MdPLATZ16 | MD03G1131600 | -- | -- | negative |
| MdPLATZ16 | MD16G1186600 | PRX2F | Peroxiredoxin-2F mitochondrial | negative |
| MdPLATZ16 | MD14G1177900 | PAT1, TRP1 | tryptophan biosynthesis 1 | negative |
| MdPLATZ16 | MD08G1147300 | ALBA2, IRP7 | Alba DNA/RNA-binding protein | negative |
| MdPLATZ16 | MD11G1285200 |  | Protein of unknown function | negative |
| MdPLATZ16 | MD11G1174600 | IQD5 | IQ-domain 5 | negative |
| MdPLATZ16 | MD10G1081000 | AT1G47740 | PPPDE putative thiol peptidase family protein | negative |
| MdPLATZ16 | MD11G1032700 | AT1G55840 | Sec14p-like phosphatidylinositol transfer family protein | negative |
| MdPLATZ16 | MD09G1003400 | AT1G08660 | MALE GAMETOPHYTE DEFECTIVE 2 | negative |
| MdPLATZ16 | MD16G1230400 | PLPP1 | Haloacid dehalogenase-like hydrolase (HAD) superfamily protein | negative |
| MdPLATZ16 | MD04G1103800 | AT5G02970.1 | alpha/beta-Hydrolases superfamily protein | negative |
| MdPLATZ16 | MD14G1152500 | RL7A2 | Ribosomal protein L7Ae/L30e/S12e/Gadd45 family protein | negative |
| MdPLATZ16 | MD10G1160700 | AT5G48660 | B-cell receptor-associated protein 31-like | negative |
| MdPLATZ16 | MD08G1244300 | RAA11C | RAB GTPase 11C | negative |
| MdPLATZ16 | MD15G1014500 | AT3G27230.1 | S-adenosyl-L-methionine-dependent methyltransferases superfamily protein | negative |
| MdPLATZ16 | MD11G1096300 | SDIR1 | RING/U-box superfamily protein | negative |
| MdPLATZ16 | MD12G1158000 | -- | -- | negative |
| MdPLATZ16 | MD15G1374400 | RPS10, US10M | ribosomal protein S10 | negative |
| MdPLATZ16 | MD02G1178200 | CCD32 | CYCLIN D3 | negative |
| MdPLATZ16 | MD10G1317700 | ADAL, MAPDA | adenosine/AMP deaminase family protein | negative |
| MdPLATZ16 | MD10G1190800 | PNK1 | pfkB-like carbohydrate kinase family protein | negative |
| MdPLATZ16 | MD11G1264700 | RAA1B | RAB GTPase homolog A1B | negative |
| MdPLATZ16 | MD16G1175900 | AT1G08820 | vamp/synaptobrevin-associated protein 27-2 | negative |
| MdPLATZ16 | MD11G1206500 | ES8Y | Ribosomal protein S8e family protein | negative |
| MdPLATZ16 | MD15G1344500 | EMB1968, RFC4 | ATPase family associated with various cellular activities (AAA) | negative |
| MdPLATZ16 | MD08G1243200 | TRAPPC2 | SNARE-like superfamily protein | negative |
| MdPLATZ16 | MD16G1257700 | RUXF | small nuclear ribonucleoprotein F | negative |
| MdPLATZ16 | MD03G1294400 | TBL6 | TRICHOME BIREFRINGENCE-LIKE 6 | negative |
| MdPLATZ16 | MD10G1078600 | MED32, MED2 | mediator of RNA polymerase II transcription subunit-like protein | negative |
| MdPLATZ16 | MD01G1118800 | KCS5 | 3-ketoacyl-CoA synthase 5 | negative |
|  |  |  |  |  |

**Supplementary Fig. S1**


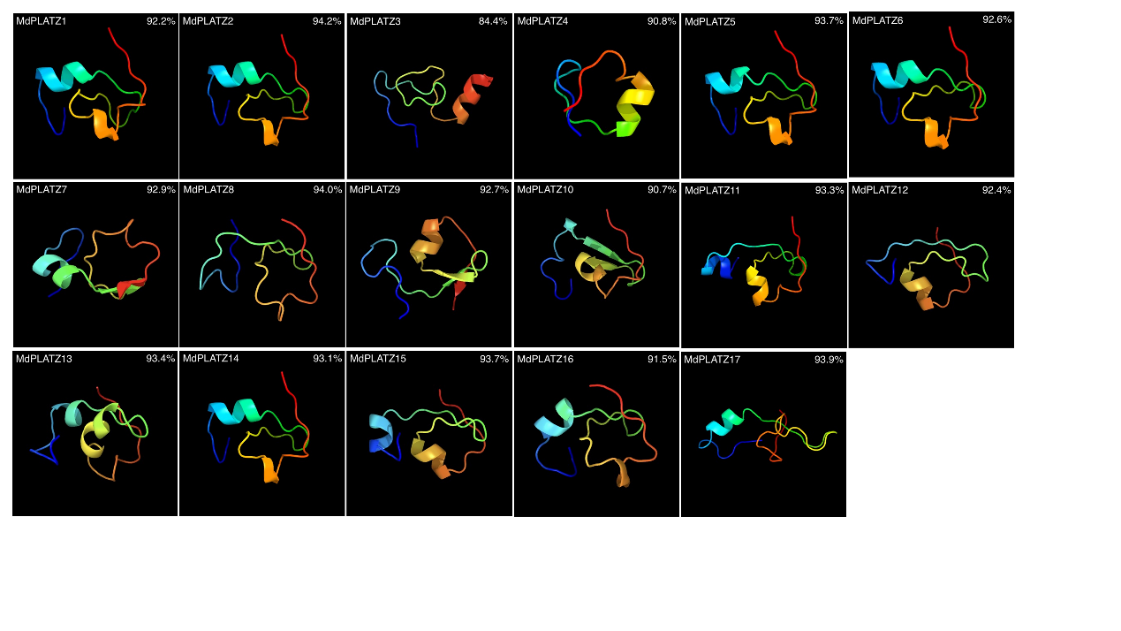


**Fig. S1 Predicted tertiary protein structures of MdPLATZ proteins.** Percentages represent credibility.

**Supplementary Fig. S2**


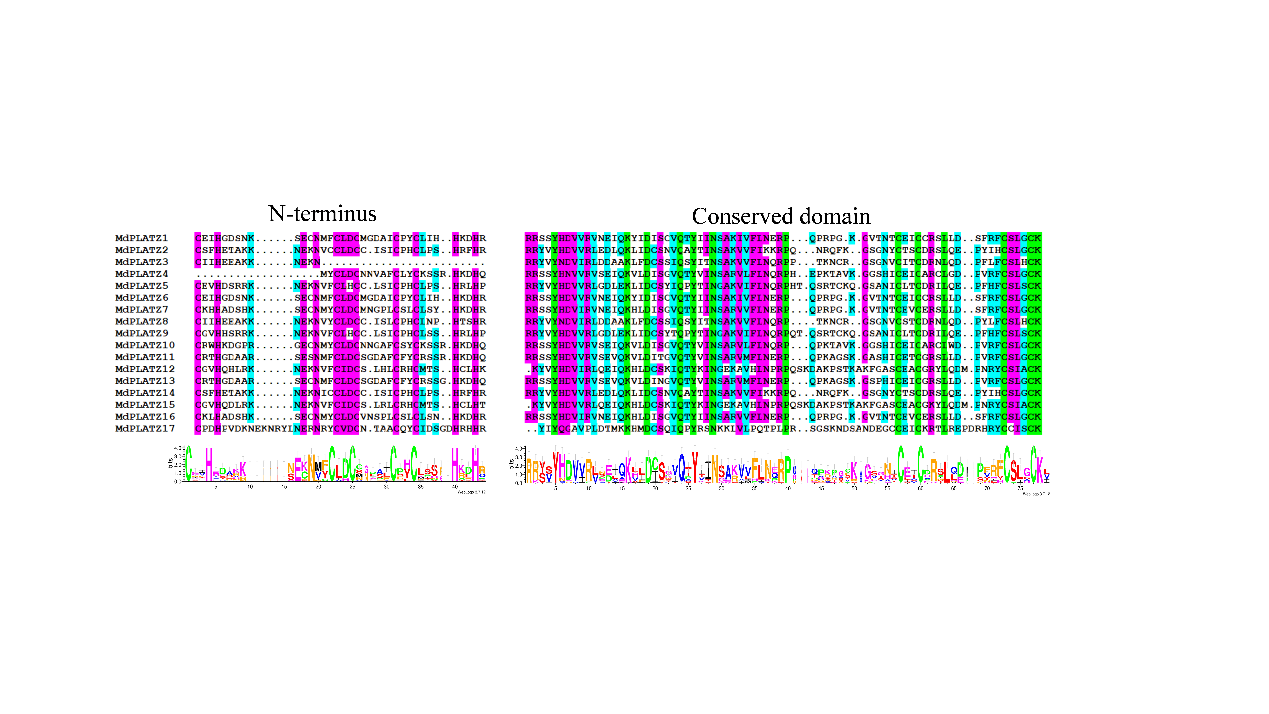
 **Fig. S2 Multiple sequence alignments of MdPLATZ proteins.**
